# The SARS‐unique domain (SUD) of SARS‐CoV and SARS‐CoV‐2 interacts with human Paip1 to enhance viral RNA translation

**DOI:** 10.15252/embj.2019102277

**Published:** 2021-04-20

**Authors:** Jian Lei, Yue Ma‐Lauer, Yinze Han, Matthias Thoms, Robert Buschauer, Joerg Jores, Volker Thiel, Roland Beckmann, Wen Deng, Heinrich Leonhardt, Rolf Hilgenfeld, Albrecht von Brunn

**Affiliations:** ^1^ Institute of Biochemistry Center for Structural and Cell Biology in Medicine University of Lübeck Lübeck Germany; ^2^ German Center for Infection Research (DZIF) Hamburg–Lübeck– Borstel–Riems Site University of Lübeck Lübeck Germany; ^3^ State Key Laboratory of Biotherapy and Cancer Center National Clinical Research Center for Geriatrics West China Hospital Sichuan University Chengdu China; ^4^ Max‐von‐Pettenkofer Institute Ludwig‐Maximilians‐University Munich Munich Germany; ^5^ German Center for Infection Research (DZIF) Munich Germany; ^6^ Gene Center Munich Department of Biochemistry Ludwig‐Maximilians‐University Munich Munich Germany; ^7^ Institute of Veterinary Bacteriology Vetsuisse Faculty University of Bern Bern Switzerland; ^8^ Institute of Virology and Immunology University of Bern Bern Switzerland; ^9^ Department of Biology and Center for Integrated Protein Science Ludwig‐Maximilians‐University Munich Planegg‐Martinsried Germany; ^10^ College of Veterinary Medicine Northwest A&F University Yangling Shaanxi China; ^11^ Institute of Molecular Medicine University of Lübeck Lübeck Germany

**Keywords:** coronavirus, eukaryotic translation initiation factors, macrodomain, protein synthesis, virus‐host interactions, Microbiology, Virology & Host Pathogen Interaction, Protein Biosynthesis & Quality Control, Structural Biology

## Abstract

The ongoing outbreak of severe acute respiratory syndrome (SARS) coronavirus 2 (SARS‐CoV‐2) demonstrates the continuous threat of emerging coronaviruses (CoVs) to public health. SARS‐CoV‐2 and SARS‐CoV share an otherwise non‐conserved part of non‐structural protein 3 (Nsp3), therefore named as “SARS‐unique domain” (SUD). We previously found a yeast‐2‐hybrid screen interaction of the SARS‐CoV SUD with human poly(A)‐binding protein (PABP)‐interacting protein 1 (Paip1), a stimulator of protein translation. Here, we validate SARS‐CoV SUD:Paip1 interaction by size‐exclusion chromatography, split‐yellow fluorescent protein, and co‐immunoprecipitation assays, and confirm such interaction also between the corresponding domain of SARS‐CoV‐2 and Paip1. The three‐dimensional structure of the N‐terminal domain of SARS‐CoV SUD (“macrodomain II”, Mac2) in complex with the middle domain of Paip1, determined by X‐ray crystallography and small‐angle X‐ray scattering, provides insights into the structural determinants of the complex formation. *In cellulo*, SUD enhances synthesis of viral but not host proteins via binding to Paip1 in pBAC‐SARS‐CoV replicon‐transfected cells. We propose a possible mechanism for stimulation of viral translation by the SUD of SARS‐CoV and SARS‐CoV‐2.

## Graphical abstract

Structural and functional analysis of the association of the Nsp3 SUD domain with the host translation regulator Paip1 reveals its role in promoting viral but not host protein synthesis in SARS‐CoV‐infected cells.

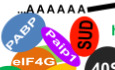

## Introduction

Coronaviruses (CoVs) are enveloped positive‐sense single‐stranded RNA (+ssRNA) viruses belonging to the subfamily *Coronavirinae* within the family *Coronaviridae* of the order *Nidovirales*. They comprise four genera: *alpha‐*, *beta‐*, *gamma‐,* and *delta‐coronavirus* (Adams & Carstens, 2012). Severe acute respiratory syndrome coronavirus (SARS‐CoV) and Middle‐East respiratory syndrome coronavirus (MERS‐CoV), from the genus *betacoronavirus*, cause severe pneumonia in humans. Both connect with high case/fatality rates. In December 2019, another *beta‐*CoV (SARS‐CoV‐2, GenBank: MN908947) emerged and spread globally (Wu *et al,* 2020). SARS‐CoV‐2 leads to the disease called COVID‐19. The whole‐genome identity between SARS‐CoV and SARS‐CoV‐2 is about 80% (Zhou *et al,* 2020). In the case of SARS‐CoV, it is generally agreed that the 2003 epidemic was caused by a zoonotic transmission of a bat CoV to humans, via an intermediate host such as the civet cat (Guan *et al,* 2003). In case of MERS‐CoV, dromedary camels have been shown to act as a reservoir (Reusken *et al,* 2013), whereas the relation to similar bat CoVs (e.g., HKU4, HKU5) remains enigmatic at this point. However, the exact zoonotic pathway is still unclear for SARS‐CoV‐2. The host range of zoonotic RNA viruses is not exclusively determined by the affinity to specific receptors on the cell surface, but also by the ability of the viruses to counteract the antiviral response exerted by the innate immune system of the host. Hijacking essential host‐cell functions such as protein synthesis and using them for their benefit also pose intriguing capabilities of the viruses. To assess the zoonotic potential of animal RNA viruses, it is important to characterize such virus–host interactions at molecular detail. In the current study, we investigate the association between a portion of SARS‐CoV as well as SARS‐CoV‐2 non‐structural protein 3 (Nsp3), i.e., the “SARS‐unique domain” (SUD), and components of the translation initiation complex including poly(A)‐binding protein (PABP)‐interacting protein 1 (Paip1), PABP, and ribosomes of the host cell.

The SUD was proposed to only exist in SARS‐CoV when the first SARS‐CoV genome sequences were analyzed (Snijder *et al,* 2003). It contains three subdomains: the N‐terminal, the middle, and the C‐terminal domain, for short SUD‐N, SUD‐M, and SUD‐C. The former two domains each adopt a macrodomain fold, and the latter one has a frataxin‐like fold (Tan *et al,* 2009; Johnson *et al,* 2010). Regions similar to the middle and C‐terminal subdomains of SUD were found in MERS‐CoV, and the C‐terminal subdomain also exists in MHV (Chen *et al,* 2015; Kusov *et al,* 2015; Ma‐Lauer *et al,* 2016; Lei *et al,* 2018). In addition, SUD‐like domains occur in bat CoVs related to SARS‐CoV and SARS‐CoV‐2, e.g., WIV16 (Yang *et al,* 2016) or As6526 (Hu *et al,* 2017). Therefore, this region is not entirely unique for SARS‐CoV, and SUD‐N, SUD‐M, and SUD‐C have now been renamed as macrodomain II (Mac2), macrodomain III (Mac3), and domain preceding Ubl2 and PL^pro^ (DPUP), respectively (Lei *et al,* 2018). All these three regions exist in SARS‐CoV‐2 sharing an amino‐acid sequence identity of ~ 75% with SARS‐CoV SUD. Hilgenfeld and colleagues have previously shown by reverse genetics that deletion of Mac2 leads to a 65–70% reduction in SARS‐CoV replication, whereas Mac3 is absolutely essential for the activity of the SARS‐CoV replication/transcription complex (Kusov *et al,* 2015). The two‐domain protein Mac2–3 interacts with oligo(G) stretches in nucleic acids, sequences which can form G‐quadruplexes (Tan *et al,* 2007; Tan *et al,* 2009). In addition, Mac3 (and Mac3–DPUP) has been reported to bind oligo(A) (Johnson *et al,* 2010). Furthermore, Mac2–3 binds to the host E3 ligase, ring‐finger, and CHY zinc‐finger domain‐containing 1 (RCHY1) and promotes RCHY1‐mediated degradation of the antiviral protein p53. This, in turn, leads to increased replication of SARS‐CoV (Ma‐Lauer *et al,* 2016).

Using a high‐throughput yeast‐2‐hybrid approach to screen the human proteome with SUD as a bait (Pfefferle *et al,* 2011), we have previously identified Paip1, a component of the host translation machinery, as an interacting partner of SUD. Paip1 acts as a positive translation stimulator to regulate the key translation factor PABP in the host translation system (Derry *et al,* 2006). Human Paip1 has three isoforms (Martineau *et al,* 2008). The N‐terminal 112 residues of isoform 1 are partly absent in isoform 2 and completely lost in isoform 3. However, the N‐terminal PAM2 motif (PABP‐binding motif 2), the middle HEAT repeat domain (Paip1M; HEAT: Huntingtin, elongation factor 3 (EF3), protein phosphatase 2A (PP2A), and yeast TOR1), and the C‐terminal PAM1 motif are absolutely conserved between these three isoforms. The PAM2 (including a conserved region of ~ 15 amino‐acid residues) and PAM1 (containing an acidic region of ~ 25 amino‐acid residues) interact with the C‐terminal domain (PABC) and the N‐terminal RNA‐recognition motif 1 and 2 (RRM1 and 2) of PABP, respectively (Kozlov *et al,* 2001; Roy *et al,* 2002; Derry *et al,* 2006). The central HEAT repeat domain, Paip1M (219 residues), is not involved in binding to PABP; it adopts a crescent‐like shape with 10 α‐helices forming five HEAT repeats, which are interrupted by two antiparallel β‐strands forming a β‐hairpin protruding from the center of the molecule (Lei *et al,* 2011). Furthermore, Paip1 interacts with eIF‐4A (Craig *et al,* 1998). The Paip1:eIF‐4A interaction may ensure that only intact mRNAs are selected as translation templates (Craig *et al,* 1998). Paip1 also binds to eIF‐3 and further stimulates translation (Martineau *et al,* 2008, 2014).

In the present study, we found that SARS‐CoV SUD binds Paip1 in HEK‐293 cells. This interaction also exists with SARS‐CoV‐2. We report the crystal structure of Mac2 (SUD‐N) in complex with Paip1M. In addition, we show that SUD enhances viral but not host protein synthesis via interacting with Paip1 in pBAC‐SARS‐CoV replicon‐transfected cells. These findings suggest that in SARS‐CoV‐infected cells, the virus takes advantage of the host translation machinery for its own benefit via the SUD:Paip1 interaction.

## Results

### The “SARS‐unique domain” interacts with poly(A)‐binding protein‐interacting protein 1 in HEK‐293 cells

**Figure 1:**
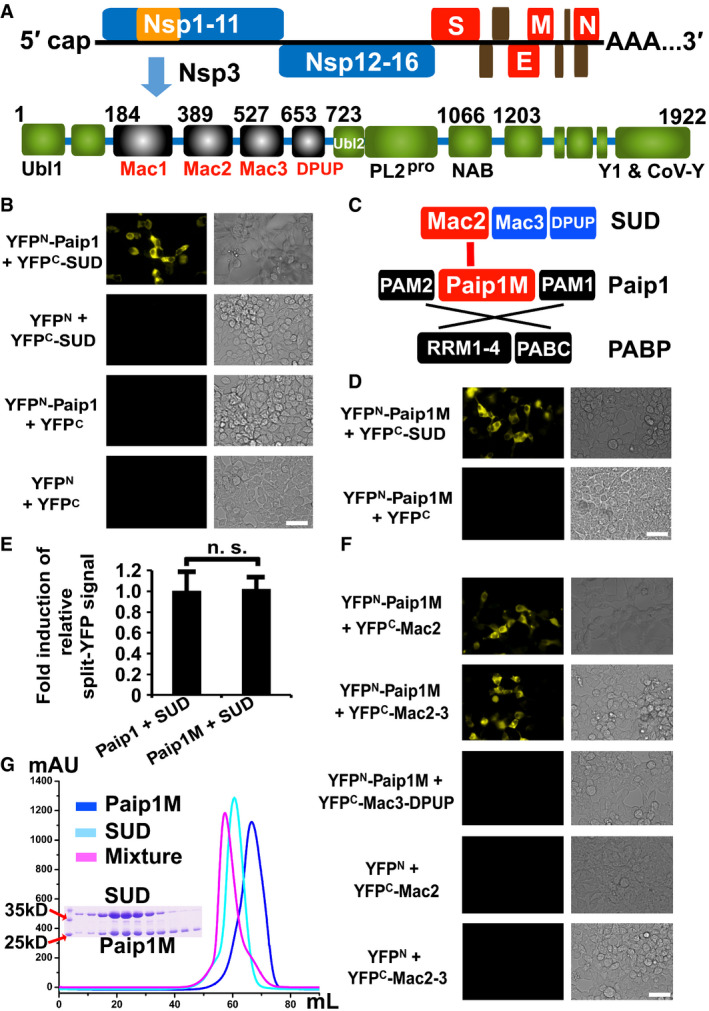
SUD interacts with Paip1 Genome organization of SARS‐CoV and schematic illustration of Nsp3 domains. DPUP: domain preceding Ubl2; Mac: macrodomain; NAB: nucleic acid‐binding domain; PL^pro^: papain‐like protease; Ubl1: ubiquitin‐like domain 1.SUD binding to Paip1 in HEK‐293 cells as demonstrated by the split‐YFP assay.Schematic presentation of SUD, Paip1, and PABP. Interactions between SUD and Paip1, as well as PAPB and Paip1 are indicated by red and black lines, respectively. PAM: PABP‐binding motif; Paip1M: the middle domain of Paip1; RRM: RNA‐recognition motif; PABC: the C‐terminal domain of PABP.Paip1M alone does interact with SUD.SUD binds to Paip1 and Paip1M with the same binding intensity. n. s.: not significant.The Mac2 subdomain is crucial for interacting with Paip1M.Paip1M and SUD interaction was confirmed *in vitro* by a gel filtration assay. Data information: Scale bars represent 50 µm.

High‐throughput yeast‐2‐hybrid (Y2H) screening of protein––protein interactions between the individual SARS‐CoV proteins and the human proteome had previously been performed by von Brunn and colleagues (von Brunn *et al,* 2007; Pfefferle *et al,* 2011), leading to the identification of many virus––host interactions. Using SUD (Fig 1A; amino‐acid residues 389–720 of SARS‐CoV Nsp3; strain: Frankfurt; GenBank: AY291315) as a bait to screen the host proteome, Paip1 (NCBI accession no. NP_877590.1) was identified as a binding partner. In order to confirm this interaction, we performed split‐YFP (yellow fluorescent protein) assays (Walter *et al,* 2004) in human cells (HEK‐293). In this assay, bait and prey proteins were fused to the N‐terminal (YFP^N^) and C‐terminal (YPF^C^) YFP fragments (or vice versa), respectively. If bait and prey proteins interact with each other, the YFP^N^ and YFP^C^ fragments will form the complete fluorescent protein and emit a yellow fluorescence when irradiated at 514 nm. When YFP^N^‐Paip1 and YFP^C^‐SUD were co‐transfected into HEK‐293 cells, the YFP signal was observed indicating binding of SUD to Paip1 in the cytosol of HEK‐293 cells (Fig 1B). For negative controls, YFP^N^‐Paip1 together with YFP^C^ or YFP^C^‐SUD together with YFP^N^ were co‐transfected. In addition to split‐YFP, the SUD:Paip1 interaction was also confirmed by a fluorescence‐3‐hybrid (F3H) assay (Herce *et al,* 2013) (Appendix Fig S1A). These results demonstrate an interaction between SUD and Paip1 *in vivo*.

### The N‐terminal SUD subdomain (SUD‐N, or Mac2) interacts with the middle domain of Paip1 (Paip1M)

We next identified the interacting regions between Paip1 and SUD in detail. As described in the introduction, Paip1 contains the N‐terminal PAM2, the middle domain (Paip1M), and the C‐terminal PAM1 (Fig 1C). The small PAM2 (about 15 residues) and PAM1 (about 25 residues) domains interact with the C‐terminal PABC domain of PABP and the N‐terminal RRM1/2 of PABP, respectively (Fig 1C; Roy *et al,* 2002; Derry *et al,* 2006). As PAM2 is also involved in binding eIF‐3 (Martineau *et al,* 2008); it is less likely that SUD interacts with Paip1 through these two small regions as well. The middle domain of Paip1 (Paip1M; residues Thr78‐Ser296) contains five HEAT repeats (Lei *et al,* 2011). Such motifs are often involved in protein––protein interactions (Yoshimura & Hirano, 2016). Therefore, we hypothesized that Paip1M is the most likely binding site for SUD. The split‐YFP assay confirmed this assumption (Fig 1D). Furthermore, quantification of the split‐YFP signal showed that the intensities of the signal for Paip1:SUD and Paip1M:SUD are almost identical (Fig 1E), suggesting that other parts of Paip1 do not contribute to binding SUD. Moreover, an F3H assay also confirmed that Paip1M interacts with SUD in BHK cells (Appendix Fig S1).

On the other hand, SUD comprises three subdomains (Fig 1A), namely the N‐terminal Mac2 (SUD‐N; Lys389‐Leu526 [Nsp3 numbering]), the middle Mac3 (SUD‐M; Gly527‐Ser652), and the C‐terminal DPUP (SUD‐C; Lys653‐Ser720; see Lei *et al,* 2018, for a review). We prepared different constructs of SUD, including Mac2, Mac2–3, and Mac3–DPUP, to test the binding capability with Paip1M by using the split‐YFP and F3H assays. Both Mac2 and Mac2–3 interact with Paip1M, while Mac3–DPUP does not (Fig 1F and Appendix Fig S1B). These results demonstrate that Mac2 is essential for binding Paip1M.

We further confirmed the interaction results by gel filtration. Paip1M and SUD were purified separately. Subsequently, these two proteins were mixed with a molar ratio of about 1.2:1 (overnight incubation). The mixture was subjected to size‐exclusion chromatography. The peak of this mixture was clearly shifted compared to the peak position of each Paip1M or SUD (Fig 1G). Next, we investigated the peak shift in size‐exclusion chromatography when Mac2, Mac2–3, Mac3, or Mac3–DPUP was mixed with Paip1M. The gel filtration results showed that Mac2 and Mac2–3 but not Mac3 or Mac3–DPUP with Paip1M displayed a peak shift when compared to the corresponding peak of each single protein (Appendix Fig S2). Taken together, these results indicate that only Mac2 binds Paip1M.

### Crystal structure of the binary complex Mac2:Paip1M

In order to elucidate the structural basis of the SUD:Paip1M interaction by X‐ray crystallography, we tried to crystallize SUD:Paip1M, Mac2–3:Paip1M, and Mac2:Paip1M. These attempts only yielded crystals for the latter complex. However, the diffractive power of the Mac2:Paip1M crystals was poor. After trying many optimization methods and testing over 240 crystals at the synchrotron, we managed to collect a 3.5‐Å dataset. Determination of the structure of this complex by molecular replacement failed (search models: Mac2 [PDB entry: 2W2G, Tan *et al,* 2009] or Paip1M [PDB entry: 3RK6, Lei *et al,* 2011]). Next, we prepared SeMet‐Mac2 and grew crystals of its complex with native Paip1M. The diffraction from these crystals was even worse; after testing more than 40 crystals, 5.3‐Å single‐wavelength anomalous dispersion (SAD) data were collected. However, ~ 5‐Å diffraction data contain, in principle, sufficient information to determine the true structure (Schröder *et al,* 2010). The structure of the Mac2:Paip1M complex was finally determined by first using the SAD data to position Mac2 in the asymmetric unit and then employing molecular replacement to locate Paip1M. Crystallographic statistics are presented in Appendix Table S1.

**Figure 2:**
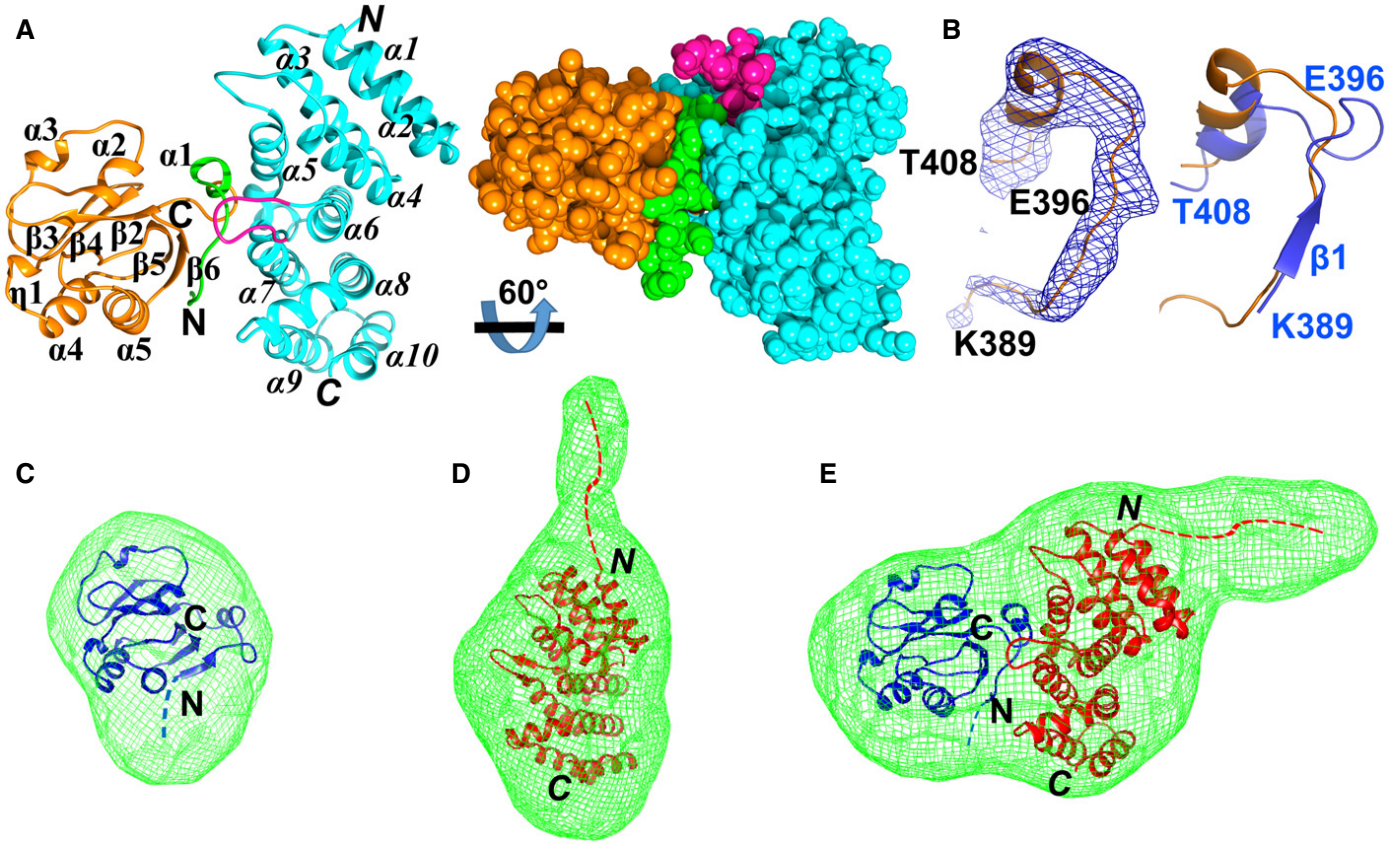
The overall structure of Mac2 in complex with Paip1M ALeft image: crystal structure of the complex between Mac2 (orange) and Paip1M (light blue). Mac2 possesses a three‐layer α/β/α fold. α1, 2, and 3, as well as η1 and α4, 5 form the two outside layers, whereas the β sheet (β3–β4‐β2–β5–β6) forms the central layer. The first 16 residues of Mac2 are shown in green. Paip1M comprises 10 helices within two layers. α1, 3, 5, 7, and 9 form one layer while α2, 4, 6, 8, and 10 form the other. The long loop region (Ile204–Thr212) is displayed in purple. The N and C termini of Mac2 and Paip1M (in *italics*) are marked. Right image: space‐filling representation showing the tight interaction between Mac2 and Paip1M.BLeft image: F_o_–F_c_ difference density omit map (2.5σ; blue) for the region Lys389–Thr408 of Mac2 (orange) in this complex. Right image: conformational change of this region between free Mac2 (blue; PDB entry: 2W2G, Tan *et al,* 2009) and Mac2 in this complex.C–EThe crystal structures of Mac2 (C; blue), Paip1M (D; red; PDB entry: 3RK6, Lei *et al,* 2011), and Mac2:Paip1M (E) fit the shape of envelopes derived from the small‐angle X‐ray scattering (SAXS) data. The blue and red dashed lines indicate the four (GSHM) and seventeen (GSHMASMTGGQQMGRGS) artificial residues remaining from the expression vectors in Mac2 and Paip1M, resp. Data information: Images A and C–E were prepared by using Chimera (Pettersen *et al,* 2004). Image B was prepared with PyMOL (Schrödinger; http://www.pymol.org).

The overall structure of the Mac2:Paip1M complex is displayed in Fig 2A. One Mac2:Paip1M heterodimer exists per asymmetric unit. Mac2 adopts the typical α/β/α macrodomain fold (Fig 2A, left image). According to the DSSP server (Kabsch & Sander, 1983), the order of the secondary structure elements of Mac2 is α1–β2–α2–α3–β3–β4–η1–α4–β5–α5–β6 (η: 3_10_ helix). A central β sheet with five predominantly parallel β strands (β3–β4–β2–β5–β6; only β3 is antiparallel) is flanked by α1, α2, and α3 on one side, and η1, α4, α5 on the other side. The root‐mean‐square difference (RMSD) between Mac2 in the complex and in Mac2‐Mac3 (PDB entry 2W2G, chain A; Tan *et al,* 2009) is 0.6 Å for the corresponding Cα atoms using the Align program (Satow *et al,* 1986). The main structural difference between the two Mac2 structures is located within the N‐terminal segment Lys389–Thr408. This region includes strand β1 and helix α1 in free Mac2–3 (Tan *et al,* 2009). However, the β‐strand is replaced by a loop in the Mac2:Paip1M complex (Fig 2B).

Paip1M displays the expected HEAT repeat fold, with 10 helices being the dominant secondary structure elements (Fig 2A). The RMSD is 1.1 Å between Paip1M in the complex and the free protein (PDB entry 3RK6, chain B, Lei *et al,* 2011). An obvious conformational change exists in the region Ile204‐Thr212. In the Mac2:Paip1M complex, this region is a long loop (Fig 2A), while it forms a β‐hairpin in free Paip1M (Lei *et al,* 2011). This conformational change does not come as a surprise, because we have noticed previously that this region is very flexible (Lei *et al,* 2011).

The interaction surfaces between Mac2 and Paip1M show good complementarity (Fig 2A, right image). About 840 Å^2^ of the surface of Mac2 and 779 Å^2^ of the surface of Paip1M are buried upon complex formation, according to the PDBePISA server (complex formation significance score: 0.86 on a scale from 0 to 1, Krissinel & Henrick, 2007). The predominant interaction regions between Mac2 and Paip1M are confined to the N‐terminal loop Ile394–Leu407 of Mac2 and to α5 (Arg160–Arg168), the central loop (Ile204–Thr212), and the N‐terminal half of α7 (Ala214–Glu223) of Paip1M.

### Solution structure of the binary complex

In order to investigate the Mac2:Paip1M complex in solution, we performed small‐angle X‐ray scattering (SAXS) experiments. The SAXS data were measured at five different protein concentrations each of Mac2, Paip1M, and Mac2:Paip1M. These data were subjected to Guinier analysis (Putnam, 2016). The resulting Guinier plot was used to obtain the intensity of zero‐angle scattering *I*_0_ and the radius of gyration (*R*_g_; estimate of the overall size of the particle). According to the *I*_0_ values of the standard BSA, and of the samples Mac2, Paip1M, and Mac2:Paip1M, the molar masses (MWs) of the particles in these three samples were calculated (Appendix Table S2). The MW of the particles in each sample was close to the theoretical value of the monomer, thus indicating a monomeric status for each protein in solution.

Subsequently, *ab‐initio* shape determination of each sample was performed according to the SAXS curves (program DAMMIF, Franke & Svergun, 2009). In case of Mac2, the overall shape of the SAXS‐derived model fitted well to the crystal structure of the Mac2 domain in Mac2–3 (Tan *et al,* 2009; Fig 2C). However, the SAXS‐derived model exhibited an extra region when compared to the crystal structures of Paip1M and Mac2:Paip1M (Fig 2D and E). This extension seen in the SAXS‐derived model is very likely due to the 17 residues GSHMASMTGGQQMGRGS that remained attached to the N‐terminus of Paip1M from the cloning vector pET‐28a. This artificial N‐terminal extension of Paip1M had not been included in the crystallographic model due to lack of electron density (Lei *et al,* 2011). The length of the 17 additional residues was estimated by comparison to the extended region Glu203 to Arg213 of Paip1M. In addition, the *R*_g_ value of Paip1M is 24 ± 1 Å in solution, whereas it is 19 Å when calculated from the crystal structure (program *CRYSOL*; Svergun *et al,* 1995) (Appendix Table S2). The 5 Å difference in *R*_g_ also indicates the larger size of Paip1M in solution compared to the crystal structure. In contrast, only four artificial residues (GSHM) had been added by the cloning procedure to the N‐terminus of Mac2.

In any case, the SAXS data confirm that the Mac2:Paip1M complex has a similar binding pattern in solution as in the crystal structure. On the other hand, we suspected that the artificial N‐terminal 17‐residue extension of Paip1M, which is quite flexible, may be detrimental for obtaining good crystals. Therefore, a new construct of Paip1M with only four extra residues (GSHM) was prepared. However, the diffraction from the crystals formed by Paip1M thus modified in complex with Mac2 was not improved.

### The N‐terminal region Lys389–Thr404 of Mac2 is necessary for binding Paip1M

**Figure 3:**
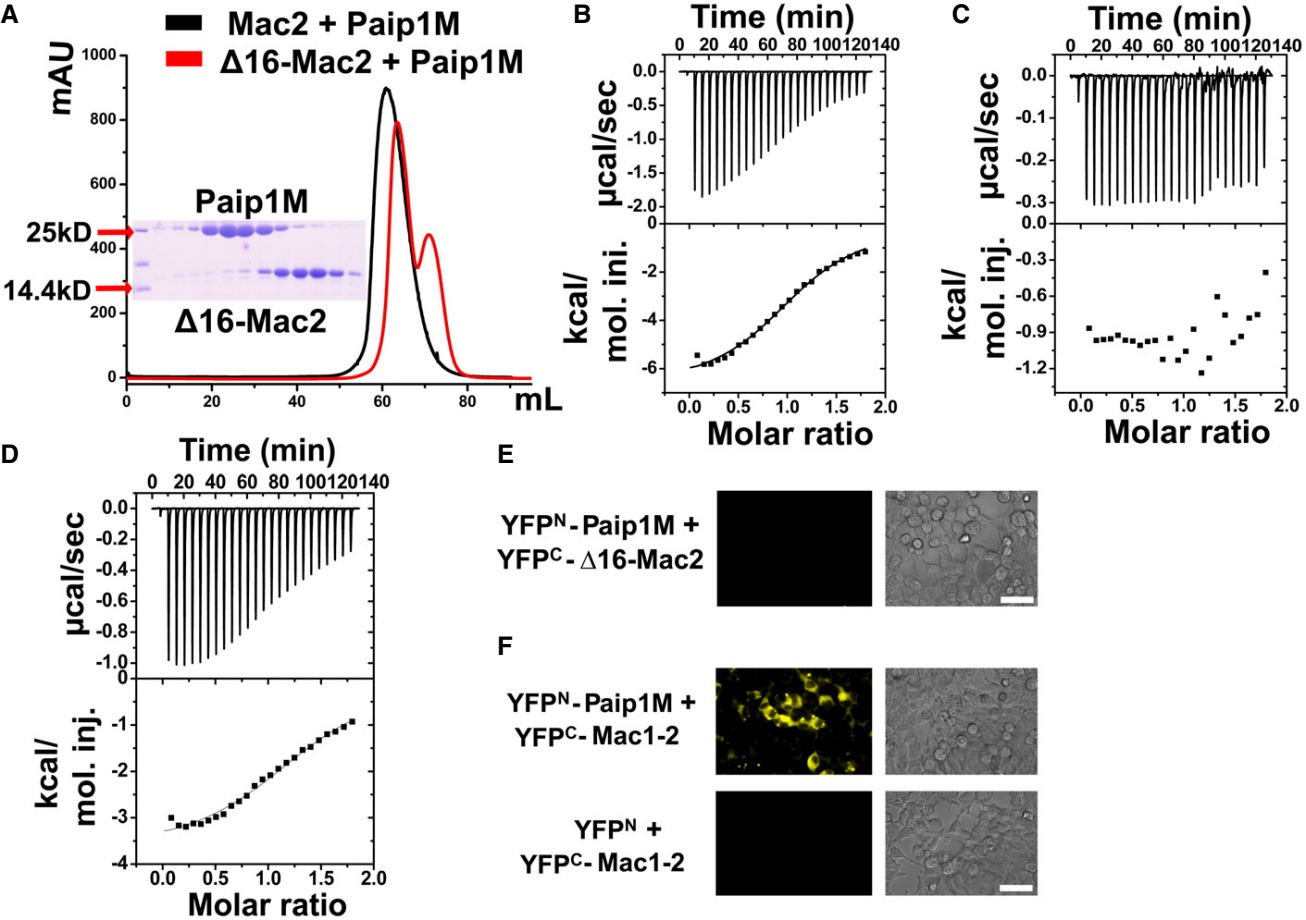
The N‐terminal 16 residues of Mac2 are important for Paip1M binding and Mac1 does not affect Mac2:Paip1 interaction AThe Mac2 and Paip1M interaction was impaired by removing the N‐terminal 16 residues as revealed by the gel filtration assay.BIsothermal titration calorimetry (ITC) assay of Mac2 binding Paip1M. The raw calorimetric curve is displayed on top while the fitted binding isotherm curve is at the bottom. The measured values are as follows: *N* = 0.94 ± 0.01 (binding stoichiometry); *K*_d_ = 15.5 ± 1.5 μM (dissociation constant); Δ*H* = −6,778 ± 149 cal/mol (standard molar enthalpy change for binding); Δ*S* = −0.716 cal/mol/K (standard molar entropy change).CΔ16‐Mac2 lost all binding affinity to Paip1M in the ITC experiment.DITC result of Mac1–2 interacting with Paip1M. The experimental results are as follows: *N* = 1.14 ± 0.02; *K*_d_ = 18.6 ± 2.5 μM; Δ*H* = −3,730 ± 100 cal/mol; Δ*S* = 9.14 cal/mol/K.E, FΔ16‐Mac2 cannot bind Paip1M, Mac1–2 binds to Paip1M in the split‐YFP assay. Scale bars represent 50 µm.

Due to the low resolution of the Mac2:Paip1M crystal structure, it is difficult to determine the exact interactions between Mac2 and Paip1M; however, we observed that mainly the N‐terminal region of Mac2 is in contact with Paip1M. In order to further confirm whether this region is essential for the interaction between the two proteins, we deleted the N‐terminal 16 amino‐acid residues of Mac2 (Lys389–Thr404; this region is shown in green in Fig 2A and contains the segment from the N‐terminus to the end of the first α‐helix). Named Δ16‐Mac2, this protein failed to form a complex with Paip1M according to the gel filtration assay (Fig 3A). Furthermore, we determined the binding affinity and stoichiometry between Paip1M and Mac2 or Δ16‐Mac2 by isothermal titration calorimetry (ITC) experiments (Fig 3B and C). The dissociation constant (*K*_d_) between Mac2 and Paip1M was determined as 15.5 ± 1.5 μM, and the binding stoichiometry was 0.94 ± 0.01 with a single‐site interaction between the two proteins. A similar range of *K*_d_ values between other host and coronavirus proteins (such as human ISG15 and papain‐like protease, *K*_d_ is about 8.6 μM) was reported before (Shin *et al,* 2020). On the other hand, Δ16‐Mac2 showed no interaction with Paip1M (Fig 3C). The split‐YFP and F3H assays confirmed that neither Δ16‐SUD nor Δ16‐Mac2 bind Paip1M in HEK‐293 and BHK cells, respectively (Fig 3E and Appendix Fig S3A). Taken together, the N‐terminal Lys389–Thr404 region of Mac2 is indispensable for the interaction with Paip1M.

### Mac1, the domain preceding Mac2, does not affect the interaction between Mac2 and Paip1M

Mac2 is located between the X domain (also called macrodomain I, Mac1) and Mac3 (SUD‐M) of Nsp3 (Fig 1A, Lei *et al,* 2018). Considering the important role of the N‐terminal region of Mac2 in binding Paip1M, the question arises whether the preceding domain, Mac1, affects the Mac2 interaction with Paip1M. We prepared the Mac1–2 construct and performed the ITC assay with Mac1–2 and Paip1M (Fig 3D). The *K*_d_ value was found to be about 18.6 ± 2.5 μM, with a single binding site between Mac1–2 and Paip1M. This value very closely resembles that of the isolated Mac2 binding Paip1M (see above). The Mac1–2:Paip1M complex was subjected to the gel filtration (S200 column) assay, and a peak shift relative to Mac1‐Mac2 and Paip1M alone was observed (Appendix Fig S3B). The split‐YFP and F3H assays also confirmed the interaction between Mac1–2 and Paip1 *in vivo* (Fig 3F and Appendix Fig S3C). Taken together, the domain preceding Mac2 does not interrupt Mac2 binding to Paip1M.

### SUD and PABP do not compete with each other for interaction with Paip1

**Figure 4:**
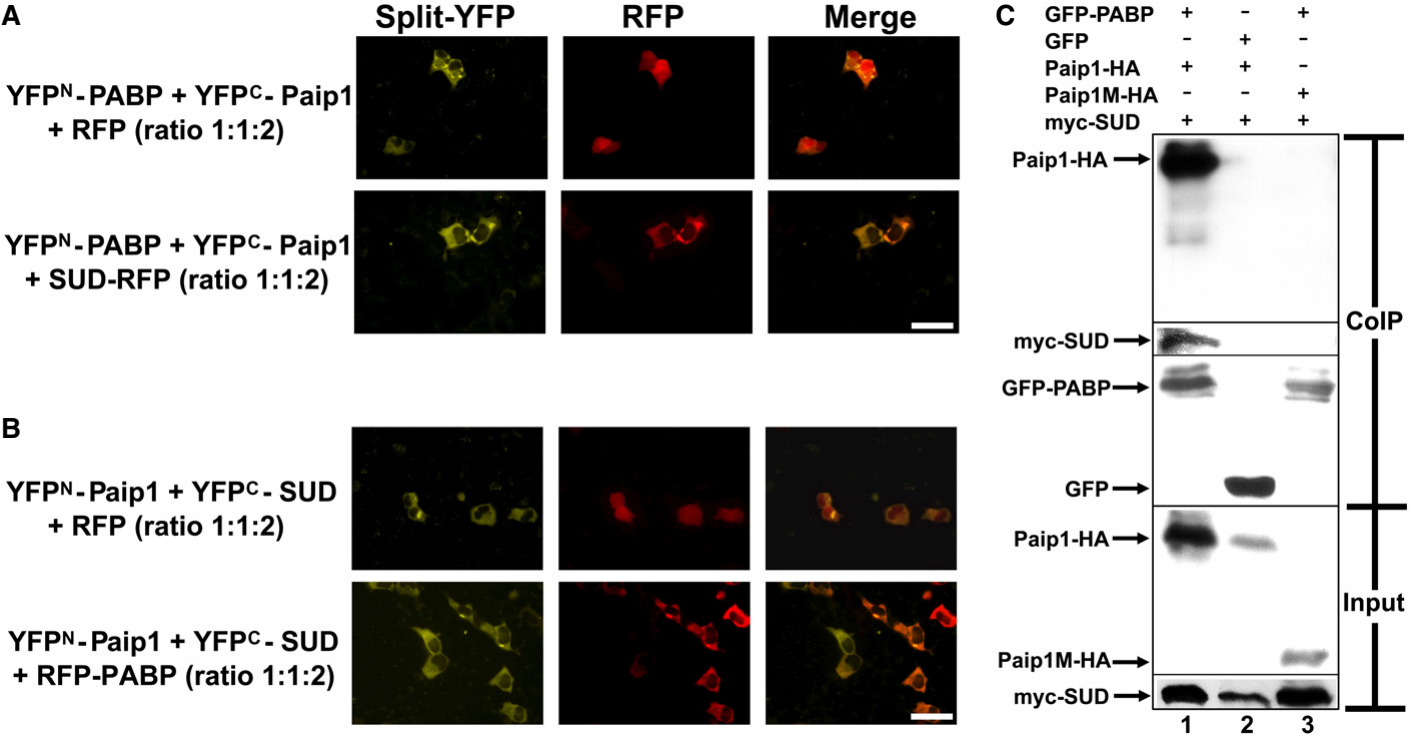
SUD, Paip1, and PABP form a ternary complex A, BSUD and PABP do not compete with each other for interacting with Paip1. (A) Excess expression of SUD‐RFP does not impair the split‐YFP signal between PABP and Paip1 (lower images), compared to the control (upper images). (B) Co‐expression of excess RFP‐PABP does not result in an impaired interaction between Paip1 and SUD either. RFP: red fluorescent protein. Scale bars represent 50 µm.CGFP‐trap‐based co‐immunoprecipitation (CoIP) assay demonstrating a complex formed by PABP, Paip1, and SUD. GFP: green fluorescent protein.

As Paip1 interacts with both SUD (Fig 1B) and PABP (Fig 1C, Craig *et al,* 1998), it is necessary to investigate whether SUD competes with PABP for interaction with Paip1. For this purpose, excess SUD‐RFP (red fluorescence protein) expressing plasmid, which corresponds to two‐fold the amount of DNA of YFP^N^‐PABP and YFP^C^‐Paip1 plasmids, were co‐transfected into HEK293 cells, and interaction of respective proteins was analyzed by split‐YFP assay. If SUD competes with PABP for interaction with Paip1, the interaction between PABP and Paip1 should be impaired in the presence of excess SUD. However, as shown in Fig 4A, co‐expression of excess SUD‐RFP does not interfere with the binding between PABP and Paip1 (lower images in Fig 4A), when compared with the control (upper images in Fig 4A). In addition, co‐expression of excess RFP‐PABP does not result in an impaired interaction between Paip1 and SUD either (Fig 4B). Therefore, we hypothesized that PABP, Paip1, and SUD might form a ternary complex.

### SUD, Paip1, and PABP form a ternary complex

To test this hypothesis, a GFP (green fluorescence protein)‐trap‐based co‐immunoprecipitation (CoIP) assay was carried out. Myc‐SUD together with Paip1‐HA (hemagglutinin‐derived tag) or Paip1M‐HA was co‐expressed with GFP‐PABP or GFP in HEK‐293 cells. Forty‐eight hours post‐transfection, cells were harvested for the GFP‐trap‐based CoIP. As shown in Fig 4C, Paip1‐HA and myc‐SUD were co‐immunoprecipitated with GFP‐PABP (Lane 1) but not with GFP alone (Lane 2), suggesting that Paip1 and SUD were co‐precipitated with GFP‐PABP due to PABP, not GFP. In addition, when Paip1M instead of full‐length Paip1 was co‐expressed, neither Paip1M nor SUD were co‐immunoprecipitated with GFP‐PABP (Lane 3). This demonstrated that there is no physical interaction between PABP and Paip1M or SUD. SUD could be pulled down by PABP only when full‐length Paip1 was simultaneously present (Lane 3 compared to Lane 1), suggesting that SUD forms a complex with PABP through Paip1.

### SUD enhances the binding affinity between Paip1 and PAPB

**Figure EV1:**
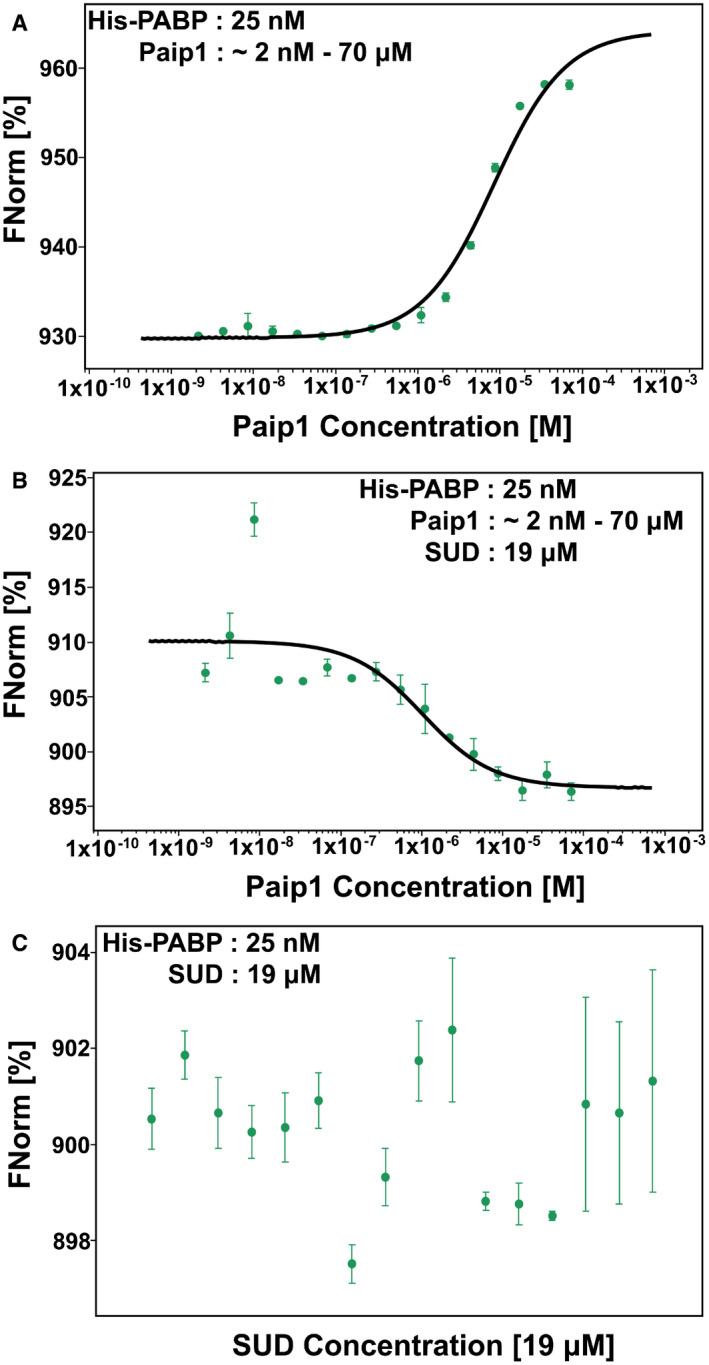
SUD enhances the interaction between PABP and Paip1 Curve fit of micro‐scale thermophoresis (MST) traces for the determination of the Paip1 and PABP‐binding affinity (*K*_d_ = 8.4 ± 1.5 μM).Curve fit of MST traces for determination of the Paip1 and PABP‐binding affinity upon adding SUD (*K*_d_ = 1.9 ± 0.6 μM).Control: SUD and PABP display no binding affinity. Data information: Data are shown as the mean ± SD from two independent replicates.

In order to test whether SUD increases or decreases the binding affinity between Paip1 and PABP, we performed a micro‐scale thermophoresis (MST) assay (Fig EV1). Fluorophore‐labeled His‐PABP protein was prepared and then serially diluted. Paip1 was mixed with labeled His‐PABP. These samples were mixed thoroughly, and measurements were performed with a Monolith NT.115 instrument (NanoTemper Technologies). The *K*_d_ value of PABP‐Paip1 is about 8.4 ± 1.5 μM (Fig EV1A). When a constant concentration of SUD protein was added to newly prepared His‐PABP:Paip1 samples, the *K*_d_ value changed to 1.9 ± 0.6 μM (Fig EV1B). As a control, the respective constant concentration of SUD protein was added to His‐PABP alone. No binding affinity was detected between these proteins (Fig EV1C). Therefore, SUD increases the binding affinity between PABP and Paip1 by about 4.4‐fold under this condition.

### SUD interacts with 40S and 80S ribosomes

**Figure EV2:**
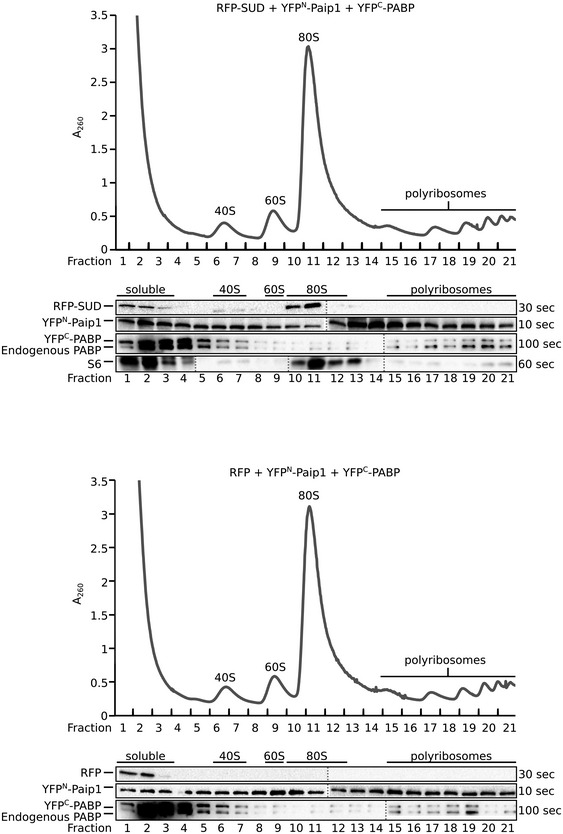
Polyribosome gradient analysis of lysates from HEK293T cells transiently transfected with either pDEST‐RFP‐SUD/pDEST‐c‐myc‐YFP^N^‐Paip1/pDEST‐HA‐YFP^C^‐PABP or pDEST‐RFP/pDEST‐c‐myc‐YFP^N^‐Paip1/pDEST‐HA‐YFP^C^‐PABP constructs The absorption at 260 nm (A_260_) and the collected fractions are shown and 40S, 60S, 80S, and polyribosome fractions are labeled. Western blot analysis of the gradient fractions using anti‐RFP, anti‐c‐myc (c‐myc‐Paip1 fusion), anti‐PABP, and rps6 (S6, 40S ribosomal subunit protein) antibodies is shown below.

Both PABP and Paip1 are components of the host translation initiation complex. As the proteins form a ternary complex with SUD and SUD enhances the binding affinity between Paip1 and PAPB, we reasoned that SUD should be found in ribosomes of the host translation machinery. We carried out polyribosome assays by transfecting HEK293T cells with plasmid constructs expressing either RFP‐SUD/c‐myc‐YFP^N^‐Paip1/HA‐YFP^C^‐PABP or RFP/c‐myc‐YFP^N^‐Paip1/HA‐YFP^C^‐PABP. Cell extracts were loaded onto linear 10–50% sucrose gradients; proteins of individual fractions were submitted to SDS–PAGE/Western blotting and probed with antibodies directed against RFP (RFP and RFP‐SUD), c‐myc (‐Paip1), PABP, and 40S ribosomal protein S6. The absorption at 260 nm (A_260_) of the collected fractions shows weak 40S and 60S and a strong 80S peak. SUD co‐elutes with 40S ribosomal protein S6 at 40S/80S ribosomes indicating the association with these ribosomal subunits (Fig. EV2). The eukaryotic small ribosomal subunit (40S) plays a central role in the initiation of translation.

### SUD stimulates viral but not host protein synthesis in pBAC‐REP‐Rluc replicon‐transfected cells

Paip1 plays an important role in regulating protein synthesis (Craig *et al,* 1998; Derry *et al,* 2006). Therefore, we tested whether SUD influences host and viral translation due to the SUD:Paip1 interaction.

**Figure 5:**
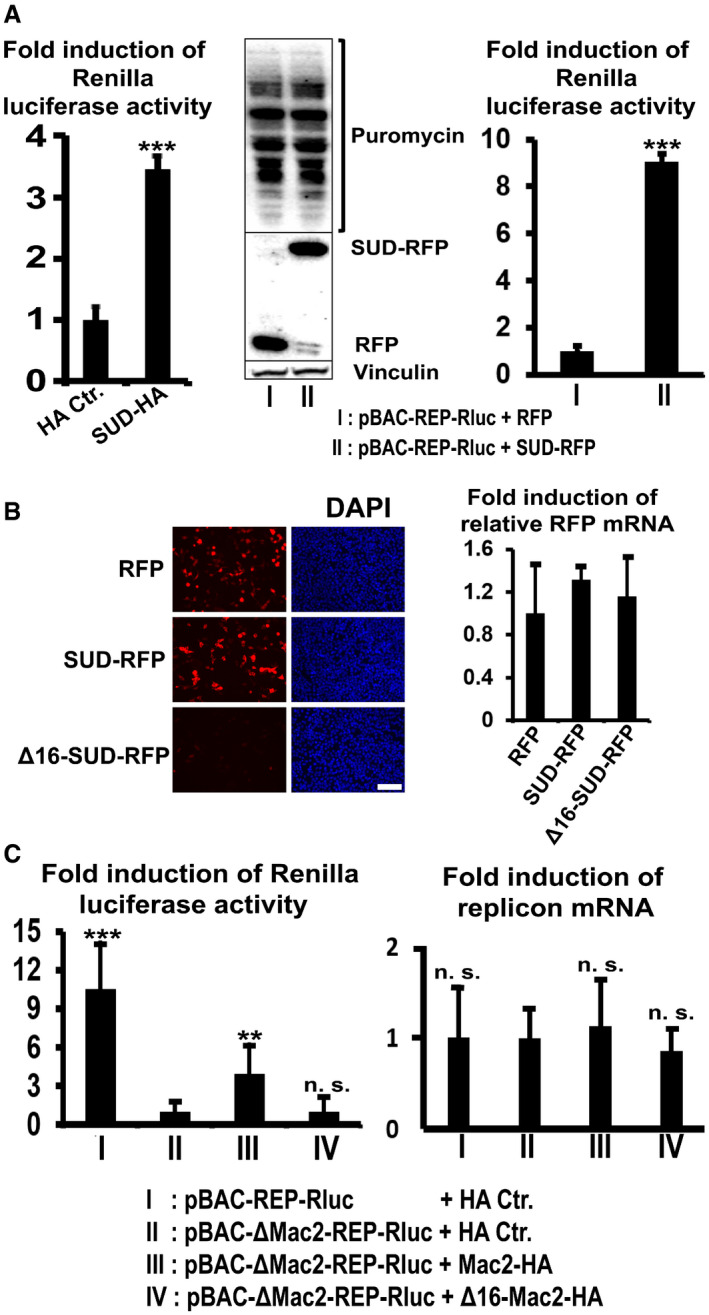
SUD generally stimulates translation but only enhances viral protein synthesis in the replicon‐transfected cells Left image: SUD generally stimulates protein translation level detected by the luciferase‐pcDNA3 reporter (*n* = 6). Middle image: SUD does not increase the amounts of total protein synthesis (host and viral proteins) in the replicon‐transfected cells. Right image: SUD augments viral protein synthesis (*n* = 8). HEK‐293 cells growing in 12‐well plates were transfected with the indicated plasmids and replicon DNA. Twenty‐four hours post‐transfection, cells were harvested for Renilla luciferase activity measurement (left and right image). For the ribopuromycylation assay, 24 h post‐transfection cells were pulsed with 3 µM puromycin for 1 h at 37°C before harvesting for Western blot analysis (middle image).Δ16‐SUD protein is not detectable but the mRNA level is normal. HEK‐293 cells were transfected with the indicated plasmids. Twenty‐four hours post‐transfection, cells were fixed for DAPI staining (left image) or lysed for a SYBR Green qPCR assay (right image, *n* = 6). Scale bars represent 100 µm.Left image: Mac2 stimulates replication of pBAC‐ΔMac2‐REP‐Rluc replicon, but Δ16‐Mac2 does not (*n* = 6). Right image: Cellular RNA was isolated for qPCR assay using primers and probes specifically recognizing SARS Nsp14 and β‐actin. The relative pBAC‐REP‐Rluc‐related replicons RNA level was calculated as the ratio of Nsp14 RNA to β‐actin RNA. *n* = 5. Data information: ***P value <0.001; **P value <0.01; n. s.: not significant. Data were analyzed using *t*‐test. Error bars represent SD.

We first examined whether SUD generally stimulates translation. To achieve this goal, a luciferase‐pcDNA3 reporter plasmid carrying Renilla luciferase under the control of the CMV (cytomegalovirus) promoter was co‐transfected with either the HA control or the SUD‐HA (C‐terminal HA) fusion plasmid to HEK‐293 cells. As a result, expression of SUD leads to induction of luciferase activity by about 2.5‐fold over the control (Fig 5A, left image) without mRNA level increase (Appendix Fig S4A). This implies that SUD generally stimulates protein synthesis.

Our next goal was to investigate whether SUD stimulates viral and/or host translation in a SARS‐CoV infection situation. To mimic normal SARS‐CoV infection, a SARS‐CoV replicon was co‐transfected with either control or SUD plasmid for examination of host and viral translation, respectively. This replicon (named pBAC‐REP‐Rluc; REP: replicon; Rluc: Renilla Luciferase) with a luciferase reporter has been constructed to mimic viral genome replication and expression (Kusov *et al,* 2015). Viral plus host protein synthesis was then tested in a ribopuromycylation assay (Schmidt *et al,* 2009; Fig 5A, middle), and viral translation alone was examined in a luciferase activity assay (Fig 5A, right) in parallel. In both assays, the pBAC‐REP‐Rluc replicon was co‐transfected with RFP control or SUD‐RFP. Interestingly, although SUD generally stimulates translation, it obviously does not enhance total protein (viral plus host protein) translation (Fig 5A, middle). However, in the presence of the SARS‐CoV replicon, the presence of SUD leads to a strong increase of the viral protein level (~ 9‐fold, Fig 5A, right), but it does not enhance host protein levels.

**Figure EV3:**
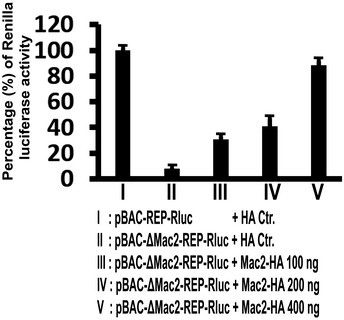
Increasing dose of Mac2 leads to increasing rescue of Renilla luciferase activity from pBAC‐ΔMac2‐REP‐RLuc The indicated replicons and plasmids were transfected into HEK293 cells using Lipofectamine 3000. Twenty‐four hours post‐transfection, cells were lysed and Renilla luciferase activity of the lysate was measured using Promega Renilla luciferase assay kit E2820. Percentage of Renilla luciferase activity was calculated as ratio of luciferase activity of pBAC‐ΔMac2‐REP‐RLuc to pBAC‐REP‐RLuc. Error bars represent SD (*n* = 6).

Subsequently, it is necessary to check whether the SUD:Paip1 interaction is the main reason for viral translation stimulation by SUD. Therefore, SUD and Δ16‐SUD were planned to be examined for their ability to rescue pBAC‐ΔSUD‐REP‐Rluc replicon expression. However, for an unknown reason, all of our different Δ16‐SUD constructs (here we only show Δ16‐SUD‐RFP and YFP^c^‐Δ16‐SUD as examples) exhibit very little expression (Fig 5B, left image and S4B, middle image) although the mRNA level is normal (Fig 5B, right image). It could be speculated that the SUD:Paip1 interaction somehow stabilizes the SUD protein. Alternatively, a rescue assay using the pBAC‐ΔMac2‐REP‐Rluc replicon together with the Mac2 or Δ16‐Mac2 constructs was carried out in order to find out the function of the SUD:Paip1 interaction for viral expression (Fig 5C), as Mac2 and Δ16‐Mac2 always have reasonable and comparable expression levels (e.g., Appendix Fig S4B middle image). The results showed that loss of Mac2, which interrupts the SUD:Paip1 interaction, leads to a ~ 10‐fold reduction of SARS replicon expression (Fig 5C, left, lane I vs II). Co‐transfection of Mac2 results in about one‐third recovery of replicon expression (Lane III vs I), while co‐expression of Δ16‐Mac2 cannot rescue the replicon expression at all (Lane IV). In this rescue assay, the RNA levels of the replicons were obviously not regulated (Fig 5C, right), suggesting that translation instead of transcription caused the difference of luciferase activity in Fig 5C (left image). To find out whether a higher dose of Mac2 could recover the Renilla luciferase activity from pBAC‐ΔMac2‐Rep‐RLuc, a dose‐dependent rescue assay was performed. As shown in Fig EV3, increasing doses of Mac2 led to an almost full rescue of the luciferase activity from pBAC‐ΔMac2‐Rep‐RLuc (Lane III to V compared with Lane I). Taken together, loss of the SUD:Paip1 interaction strongly impairs viral translation. The SUD:Paip1 interaction plays an important role for viral but not host mRNA translation in the pBAC‐REP‐Rluc‐transfected cells.

### SARS‐CoV‐2 SUD binds to Paip1

**Figure 6:**
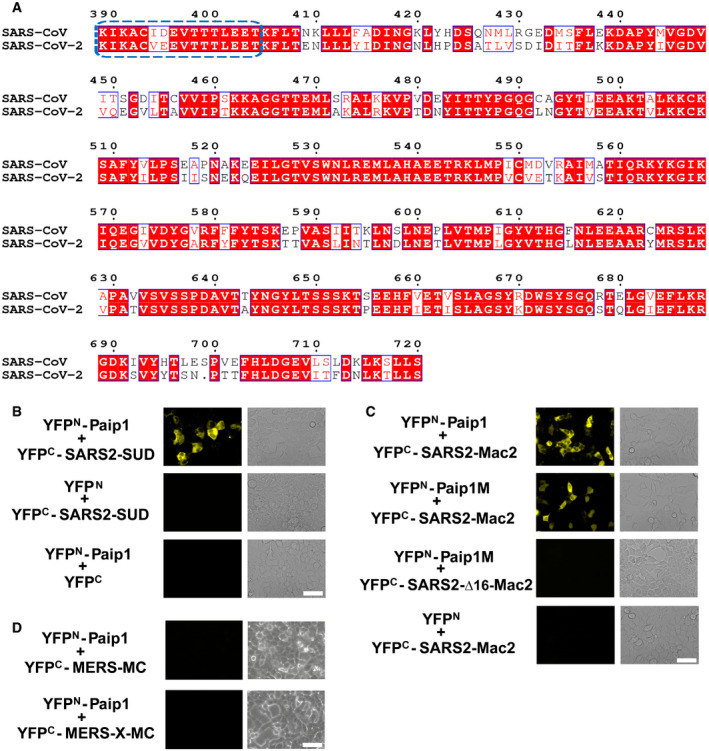
The corresponding SUD:Paip1 interaction exists in SARS‐CoV‐2 Sequence alignment of SUD between SARS‐CoV (GenBank: AY291315) and SARS‐CoV‐2 (GenBank: MN908947). Domains Mac2 (SUD‐N), Mac3 (SUD‐M), and DPUP (SUD‐C) range from K389 to L526, G527 to S652, and K653 to S720, respectively. The first 16 residues of Mac2 are boxed in blue. This figure was generated by using the ESPript server (http://espript.ibcp.fr/ESPript/ESPript/).SARS‐CoV‐2 SUD (SARS2‐SUD) interacts with Paip1 in HEK‐293 cells as demonstrated by the split‐YFP assay.SARS2‐Mac2 binds to Paip1. SARS2‐Mac2 but not Δ16‐SARS2‐Mac2 interacts with Paip1M.MERS‐CoV SUD‐MC (Mac3–DPUP) or XMC (Mac1–Mac3–DPUP) do not bind to Paip1. Data information: Scale bars represent 50 µm.

The SUD domains of SARS‐CoV and SARS‐CoV‐2 (for short, SARS2‐SUD) display an amino‐acid identity of 75% (Fig 6A) prompting us to test whether SARS2‐SUD also binds to Paip1. Not surprisingly, SARS2‐SUD and SARS2‐Mac2 interact with Paip1 (Fig 6B and C). In addition, SARS2‐Mac2 but not Δ16‐SARS2‐Mac2 bind to Paip1M (Fig 6C) implying similar functions during coronavirus translation. As MERS‐CoV does not carry a Mac‐2 domain, we further tested the binding behavior of the region corresponding to Mac3–DPUP (MERS‐MC) and Mac1–Mac3–DPUP (MERS‐X–MC) toward Paip1 (Fig 6D). We did not detect interactions of either region with Paip1.

## Discussion

So far, three emerging CoVs, SARS‐CoV, MERS‐CoV, and SARS‐CoV‐2 cause severe pneumonia in humans. Several new SARS‐like bat CoVs have been discovered in China and Korea in the past few years (Menachery *et al,* 2015; Kim *et al,* 2016; Yang *et al,* 2016; Hu *et al,* 2017). These bat CoVs use the same receptor on host cells (ACE2; angiotensin‐converting enzyme II) as SARS‐CoV or SARS‐CoV‐2 (Ge *et al,* 2013; Menachery *et al,* 2015; Yang *et al,* 2016; Hu *et al,* 2017). Hence, an outbreak of a SARS‐like coronavirus (such as SARS‐CoV‐2) can occur any time. In addition to receptor usage, the potential of a zoonotic virus to establish itself in a new host will depend on its ability to cope with the innate immune system of this host and possibly with its capability to use the host's protein synthesis machinery for its own benefit. Our study provides new insight on the latter aspect for SARS‐CoV by demonstrating that the SUD‐N (Mac2) domain interacts with the host cell translation apparatus via Paip1 and increases viral translation. This interaction may offer a new antiviral target. Importantly, the domain corresponding to SARS‐CoV SUD exists in SARS‐CoV‐2 and is identical to 75% at the amino‐acid sequence level. The N‐terminal 16 residues are almost identical between these two viruses (Fig 6A). They are required for binding to Paip1 (Fig 6C). Interestingly, the Mac2 domain is absent in MERS‐CoV (Ma‐Lauer *et al,* 2016). Only Mac1 (X domain), Mac3 (SUD‐M), and DPUP (SUD‐C) exist in this virus. Neither MERS‐CoV Mac3–DPUP (MERS‐MC) nor Mac1–Mac3–DPUP (MERS‐X–MC) interact with Paip1 (Fig 6D); therefore, the Mac2:Paip1 interaction is involved in the unique regulation of translation in SARS‐CoV and SARS‐CoV‐2 but not in MERS‐CoV. Our results help better understand the novel SARS‐CoV‐2 and other new SARS‐like CoVs that will possibly enter the human population through future zoonotic events.

**Figure 7:**
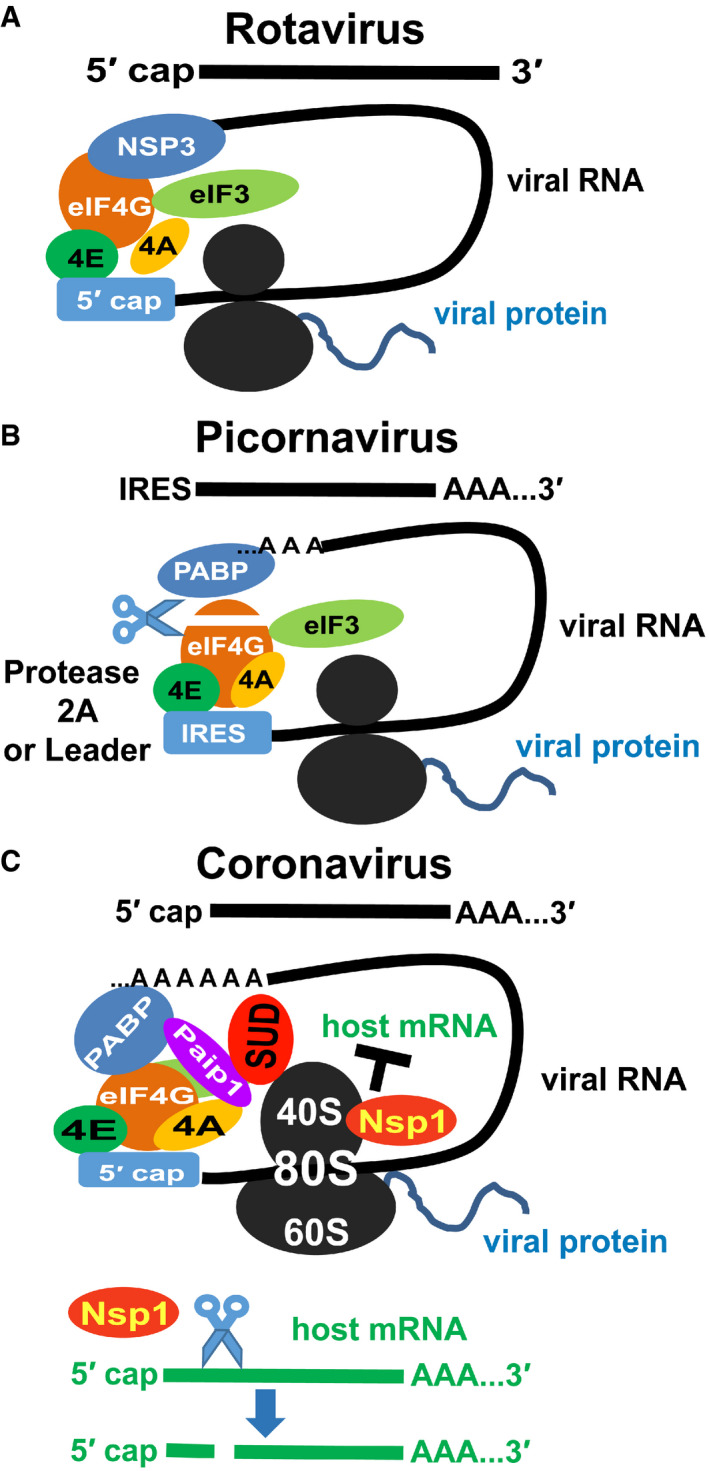
Different mechanisms of viruses regulating the host translation system Rotavirus RNAs have a 5′ cap but not a 3′ poly(A) tail. Its NSP3 protein (instead of PABP) binds to the 3′ end of the viral mRNA and interacts with eIF‐4G directly to maintain the closed‐loop RNA for the initiation of viral RNA translation and to block host mRNA circularization (Piron *et al,* 1998).Picornavirus RNA has an internal ribosome entry site (IRES) in the 5′ untranslated region and a 3′ poly(A) tail. Viral protease (2A or leader) digests eIF‐4G. The truncated eIF‐4G shows higher efficiency in the IRES‐driven translation than the cap‐dependent translation (Ohlmann *et al,* 1995; Ali *et al,* 2001).Coronavirus RNAs have a 5′ cap and a 3′ poly(A) tail. In our model, SUD associates with the 40S/80S ribosome and enhances the PABP:Paip1 interaction to stimulate the host translation machinery. Meanwhile, coronaviral Nsp1 specifically cleaves host mRNAs (green) but not viral RNAs (Kamitani *et al,* 2006; Huang *et al,* 2011). Also, viral Nsp1 blocks host mRNA binding to the 40S ribosome. As a result, SARS‐CoV could increase the viral RNA translation but inhibit host mRNA translation.

RNA viruses always seek to stimulate viral RNA translation but inhibit host protein synthesis because of the limited cellular resources as well as a means to block antiviral factor (e.g., interferon) production. However, different viruses utilize distinct strategies owing to the individual features of their mRNAs. For example, rotavirus mRNA is only 5′‐capped but not 3′‐polyadenylated (Fig 7A). Its NSP3 protein binds to the 3′ end of the viral mRNA, instead of PABP interacting with eIF‐4G, to maintain the closed‐loop RNA for the initiation of viral RNA translation and to block host mRNA circularization (Fig 7A, Piron *et al,* 1998). Differently, picornavirus RNA lacks the 5′‐cap but has a RNA stem‐loop structure in the 5′‐untranslated region (5′‐UTR). The initiation of viral RNA translation is mediated in a cap‐independent way by an internal ribosomal entry site (IRES) located in the 5′‐UTR (Fig 7B, Martínez‐Salas *et al,* 2015). The picornavirus 2A protease (or the Leader protease) cleaves eIF‐4G (Fig 7B, Lei & Hilgenfeld, 2017), and the truncated eIF‐4G works more efficiently on the viral IRES‐driven translation than on the host cap‐dependent translation (Ohlmann *et al,* 1995; Ali *et al,* 2001), thereby supporting picornavirus protein synthesis. However, coronavirus RNAs have both the 5′‐cap and the 3′‐poly(A) tail (Fig 7C), and are thus similar to the host mRNAs. Coronavirus protein synthesis involves the cap‐dependent translation machinery similar to the host (Nakagawa *et al,* 2016). Therefore, CoVs cannot use strategies similar to rotavirus or picornaviruses.

In the present study, we confirmed that SARS‐CoV Mac2 binds to the host translation stimulator, Paip1 (Fig 1F, Appendix Figs S1B and S2B). We also found that SUD (Mac2–Mac3–DPUP) regulates the mRNA translation level (Fig 5A). Like host mRNAs, the genomic and sub‐genomic RNAs of CoVs are translated in a cap‐dependent manner with the RNA cap‐to‐tail “closed‐loop” structure (Kahvejian *et al,* 2001; Derry *et al,* 2006; Nakagawa *et al,* 2016). The formation of this circular mRNA topology is the rate‐limiting initiation step of the host translation process (Kahvejian *et al,* 2001; Derry *et al,* 2006; Sonenberg & Hinnebusch, 2009; Jackson *et al,* 2010). The host elements that are involved in the initiation step are important for CoV replication. For example, Cencic *et al* (2011) reported that replication of human CoV 229E is significantly reduced by blocking the interaction between eIF‐4E and eIF‐4G. Silencing the PABP gene reduces the production of transmissible gastroenteritis CoV (TGEV) (Galán *et al,* 2009). Binding of PABP to the 3′ poly(A) of bovine CoV or mouse hepatitis CoV (MHV) is required for virus replication (Spagnolo & Hogue, 2000). PABP is a key factor by interacting with the 3′‐poly(A) tail as well as eIF‐4G to form “closed‐loop” mRNA (Kahvejian *et al,* 2001). The Paip1:PABP interaction facilitates this process and enhances the efficiency of PABP (Craig *et al,* 1998; Derry *et al,* 2006). As we mentioned above, SUD generally stimulates the mRNA translation level in HEK‐293 cells (Fig 5A, left) but only stimulates the viral translation level in the replicon‐transfected cells (Fig 5A, right). However, three questions should still be answered: (i) How does SUD enhance the translation? As we know, a strengthened Paip1:PABP interaction enhances the translation efficiency (Craig *et al,* 1998; Derry *et al,* 2006). We found that SUD enhances the binding affinity of PABP to Paip1 by about 4.4‐fold (Fig EV1), which potentially stimulates the translational level. It can be speculated that upon binding to SUD, Paip1 adopts a more favorable conformation when forming a complex with PABP. To answer this, it will be interesting to investigate the structure of SUD:Paip1:PABP in the future. (ii) Is SUD associated with the host translational apparatus and does this interaction make SUD‐N (Mac2) essential for SARS‐CoV? First, we tested the interactions between SUD and ribosomes by a polysome gradient analysis assay (Fig EV2). We find that SUD co‐elutes with a 40S ribosomal subunit marker protein (rps6) in 40S and 80S ribosomes. Initiation factors bind to this complex and facilitate scanning of messenger RNAs and initiation of protein synthesis. We hypothesize that SUD plays a major role for expression of viral mRNAs within the ternary complex with PABP and Paip1. Furthermore, we have previously answered this question in the context of a SARS‐CoV replicon where we showed that upon deletion of this domain, SARS‐CoV replication was reduced by 65–70% (Kusov *et al,* 2015). Here, we further confirmed that loss of the SUD:Paip1 interaction via Mac2 deletion leads to an about 10‐fold reduction of viral replicon activity (Fig 5C Lane II vs I), indicating that the interaction of the domain with Paip1 is critical for viral replication. (iii) The higher activity of the translation system should also increase the production of host proteins. How does SARS‐CoV manage to avoid this situation, which would be unfavorable for virus replication? SARS‐CoV Nsp1 was reported to specifically promote host but not viral mRNA degradation (Kamitani *et al,* 2006; Huang *et al,* 2011). In addition, recent studies have shown that the C‐terminus of Nsp1 from SARS‐CoV‐2 blocks the mRNA channel of 40S ribosomes (Schubert *et al,* 2020; Thoms *et al,* 2020), therefore blocking the translation of host mRNA. But mRNA carrying the corresponding viral 5′UTR can escape from translation suppression caused by SARS‐CoV and SARS‐CoV‐2 Nsp1 (Tanaka *et al*, 2012; Schubert *et al,* 2020). Therefore, during infection, the host mRNA gets degraded due to Nsp1 and host translation is blocked by the binding of Nsp1 to the mRNA channel of 40S ribosomes. However, viral RNA with viral 5′‐UTR can escape the suppression caused by Nsp1. As a final result, although SUD unselectively stimulates translation, only viral but not host translation is enhanced. This also supports our observations that SUD increases viral RNA (replicon) translation but not host gene expression in pBAC‐REP‐Rluc‐transfected cells despite the enhanced activity of the translation machinery (Fig 5A).

Based on these considerations, we propose a model to illustrate how regulation of the host translation machinery through SUD is beneficial for SARS‐CoV (Fig 7C). SUD binds to 40S/80S ribosome and enhances the interaction between Paip1 and PABP to stimulate the general translation level. Then, viral protein Nps1 not only specifically degrades the host mRNAs but also blocks host mRNA binding to the 40S ribosome, leading to the inhibition of host protein synthesis (Fig 7C). Considering that the host ribosomes interact with both viral Nsp1 (Schubert *et al,* 2020; Thoms *et al,* 2020) and SUD, it will be interesting to investigate further whether these two proteins might regulate the activity of each other.

In the past 17 years, various proteins of SARS‐CoV have been used as targets for inhibitor design (Hilgenfeld & Peiris, 2013; Hilgenfeld, 2014; Zumla *et al,* 2016), and recently, this has been expanded to SARS‐CoV‐2 (Zhang *et al,* 2020), but only Remdesivir has received limited approval for the treatment of COVID‐19. In view of the ongoing SARS‐CoV‐2 pandemic, it should be generally acknowledged now that newly emerging CoVs constitute a great threat to public health and novel approaches to drug discovery are necessary. Identification of new virus–host protein complexes as antiviral targets is a good strategy. However, we should notice that since structures of such complexes are extremely scarce, the development of antiviral approaches targeting such interactions is seriously hampered. To our knowledge, three types of structures of viral and host protein complexes are mainly available for SARS‐CoV or SARS‐CoV‐2. One of these is connected with the viral entry step: Structures of the receptor‐binding domain of the spike protein in complex with the host receptor ACE2 have been reported (Li *et al,* 2005; Yuan *et al,* 2017; Lan *et al,* 2020; Shang *et al,* 2020; Wang *et al,* 2020; Wrapp *et al,* 2020). The second type is the structure of the spike protein interacting with a human antibody (Hwang *et al,* 2006; Prabakaran *et al,* 2006; Yuan *et al,* 2020). The last type is related to the host‐cell innate immune response regulated by ubiquitin (Ub) or Ub‐like proteins, such as the structures of SARS‐CoV papain‐like protease interacting with Ub, di‐Ub, or ISG15 (interferon‐stimulated gene 15; see Lei *et al,* 2018, for a review). Recently, the structure of the SARS‐CoV‐2 papain‐like protease in complex with murine ISG15 has been reported (Shin *et al,* 2020). In addition, the cryo‐EM structures of SARS‐CoV‐2 Nsp1 with ribosomes (40S/80S) were reported (Schubert *et al,* 2020; Thoms *et al,* 2020). Here, we present the crystal structure of the SARS‐CoV Mac2:host Paip1M complex involved in the regulation of viral and host mRNA translation. More structural information on virus:host protein complexes in this field is necessary to arrive at a better understanding.

## Materials and Methods

### Recombinant production of Paip1M and SUD or SUD subdomains

The middle domain of human Paip1 (Paip1M; NCBI accession no. NP_877590.1), which comprises residues Thr78‐Ser296, was expressed in *Escherichia coli* and purified according to Lei *et al* (2011). SARS‐CoV‐2 sequences were obtained from Thao *et al* (2020).

The full‐length SUD of SARS‐CoV (strain: Frankfurt; GenBank: AY291315) comprises 332 amino‐acid residues, corresponding to residues Lys389 to Ser720 of non‐structural protein 3 (Nsp3; residue numbering starts at N‐terminus of Nsp3). It is divided into three subdomains, namely Mac2 (SUD‐N), Mac3 (SUD‐M), and DPUP (SUD‐C) (Tan *et al,* 2009; see Lei *et al,* 2018 for the new names of the three subdomains). SUD, Mac2, Mac2–3 (SUD‐NM), Mac3, and Mac3–DPUP (SUD‐MC) constructs of SARS‐CoV correspond to residues Lys389 to Ser720, Lys389 to Leu526, Lys389 to Ser652, Gly527 to Ser652, and Gly527 to Ser720 of Nsp3, respectively. Mac2 with the N‐terminal 16 residues deleted (Δ16‐Mac2) comprises residues Lys405 to Leu526. All the corresponding DNA constructs mentioned above were amplified by polymerase chain reaction (PCR) using the oligonucleotide primers specified in Appendix Table S2. The primers for all the constructs contain NdeI and XhoI cleavage sites. The digested SUD, Mac2–3 (SUD‐NM), Mac3, and Mac3–DPUP (SUD‐MC) PCR products were ligated into pET‐15b (Novagen), respectively; Mac2 (SUD‐N) and Δ16‐Mac2 were ligated into pET‐28a (Novagen). Both vectors contain an N‐terminal hexa‐histidine (His_6_) tag and a thrombin cleavage site; also, these two vectors give rise to 4 extra residues (GSHM) at the N‐terminus of the target protein after using thrombin to remove the His‐tag.

The X domain (also named Mac1) plus Mac2 (Mac1–2) construct includes residues Glu182 to Leu526 of Nsp3. The two primers for this construct contain BamHI and XhoI cleavage sites (Appendix Table S2). The digested Mac1–2 PCR product was ligated into pET28a, which leaves 17 extra residues (GSHMASMTGGQQMGRGS) at the N‐terminus of the Mac1–2 protein.

The pET15b‐ and pET28a‐related recombinant plasmids mentioned above were verified by sequencing (MWG Eurofins). Correct plasmids were transformed into *E. coli* strain Tuner (DE3; Novagen). Bacteria were incubated at 37°C overnight in 50 ml LB medium with ampicillin (50 μg/ml)/chloramphenicol (34 μg/ml) and kanamycin (25 μg/ml)/chloramphenicol (34 μg/ml), respectively. Pre‐cultures were inoculated into 2 l LB medium after 12 h. When the OD_600_ of the 2‐l culture reached about 0.8, overexpression of the target gene was induced for 8 h with 0.5 mM isopropyl‐d‐thiogalactoside (IPTG) at 30°C. Then, cultures were centrifuged for 25 min at 7,300 *g* and 4°C. Subsequently, the collected cells were resuspended in 30 ml buffer A (20 mM Tris–HCl pH 8.5, 500 mM NaCl, and 10 mM imidazole) and lysed by sonication on ice. Lysates were centrifuged for 1 h at 48,384 *g* and 4°C to remove the cell debris. Supernatants were applied to a HisTrap™ nickel column (GE Healthcare), and the His‐tagged proteins were eluted with buffer B (20 mM Tris–HCl pH 8.5, 500 mM NaCl, and 500 mM imidazole) using a linear gradient. In order to remove the His‐tag, thrombin (Sigma‐Aldrich) was added to the purified proteins overnight at 4°C. The next day, target proteins were again purified by nickel column chromatography to remove the uncleaved His‐tagged protein. All flow‐through proteins (without His‐tag) from the nickel column were subsequently purified by gel filtration (HiLoad™ 16/60 Superdex 75 column, GE Healthcare) using buffer C (20 mM Tris–HCl pH 8.5, 150 mM NaCl). The quality of target proteins was inspected by SDS–PAGE.

Furthermore, selenomethionine (SeMet) SUD‐N (Mac2) protein was prepared. The Mac2‐pET28a plasmid was transformed into *E. coli* strain SoluBL21™ (Genlantis). Transformed cells were grown in 1 l M9 medium at 37°C. When the OD_600_ of the 1‐l culture had reached 0.8, l‐selenomethionine and six other amino acids (lysine, threonine, phenylalanine, leucine, isoleucine, and valine) were added to the culture. After 15 min, Mac2 gene expression was induced for 8 h at 30°C by the addition of ITPG (final concentration 0.5 mM). The remaining steps (e.g., culture harvesting and protein purification) were done as described above.

### Complex formation and gel filtration assay

Purified Paip1M was mixed with SUD, Mac2–3, Mac2, Mac3, Mac3–DPUP, or Δ16‐Mac2 proteins. The molar ratio of Paip1M to SUD was 1.2:1, while the molar ratio of Paip1M to the other constructs (Mac2–3, Mac2, Mac3, Mac–DPUP, or Δ16‐Mac2) was 1:1.2. Mixtures were incubated at 4°C overnight. Subsequently, they were purified by gel filtration (HiLoad™ 16/60 Superdex 75 column, GE Healthcare) using buffer C. The peak position of each binary mixture was compared to the peaks of the corresponding single proteins to detect whether or not a complex was formed. The result was further confirmed by SDS–PAGE. The peak position of the Δ16‐Mac2 and Paip1M mixture was compared to the peak of the Mac2:Paip1M complex.

Differently, the mixture of Mac1–2 (X domain plus SUD‐N) and Paip1M (molar ratio = 1:1.2) was purified by Superdex 200 column chromatography (GE Healthcare).

### Crystallization and diffraction data collection

The purified complexes SUD:Paip1M, Mac2–3:Paip1M, and Mac2:Paip1M were concentrated to about 10 mg/ml in buffer C. Crystallization trials for each complex were performed at 289 K by employing the sitting‐drop vapor‐diffusion method with 0.25 µl of protein and 0.25 µl of reservoir using a Phoenix crystallization robot (Art Robbins). The following commercially available screens were used: Index™, SaltRx™ 1 & 2, Crystal Screen™ 1 & 2, PEG/Ion Screen™ 1 & 2, and PEG Rx™ 1 & 2 (Hampton Research), as well as Protein Complex Suite (Qiagen), Protein Complex Kit, and Low‐ionic Strength Kit (Sigma). Crystals were only obtained for Mac2:Paip1M under several conditions: Protein Complex Suite no. 14 (0.1 M calcium acetate, 0.1 M sodium acetate pH 4.5, 10% PEG 4000) and no. 25 (0.15 M (NH_4_)_2_SO_4,_ 0.1 M Tris pH 8.0, 15% PEG 4000), Protein Complex Kit no. 9 (0.1 M (NH_4_)_2_SO_4_, 0.1 M HEPES pH 7.5, 15% PEG 3350), and low‐ionic strength no. 21 (0.05 M MES‐Na pH 6.0, 4% PEG 3350). After optimization of the crystallization under all conditions listed above, the best crystals were obtained within one day from 0.1 M (NH_4_)_2_SO_4_, 0.1 M HEPES pH 7.3, 18% PEG 3350, 15% glycerol. These crystals were shock‐cooled in liquid nitrogen.

Diffraction of X‐rays by these crystals was poor and anisotropic. We tried many optimization methods (e.g., various crystallization temperatures, additives, and dehydration of the crystals); different cryo‐protectants were also tested. Over 240 crystals were tested at beamline 14.2 of the BESSY II synchrotron (Berlin, Germany) and beamline P11 of PETRA III, DESY (Hamburg, Germany). Finally, a native dataset to 3.5 Å was collected at a wavelength of 0.9184 Å at BESSY. However, the structure determination of this complex by molecular replacement (MR) failed despite the availability of the X‐ray structures of the isolated components, Mac2 (PDB entry: 2W2G, Tan *et al,* 2009) and Paip1M (PDB entry: 3RK6, Lei *et al,* 2011). Therefore, SeMet‐Mac2 protein was prepared. Crystals of Paip1M:SeMet‐Mac2 were obtained under the same crystallization conditions used for the native protein complex, 0.1 M (NH_4_)_2_SO_4_, 0.1 M HEPES pH 7.3, 18% PEG 3350, 15% glycerol. Diffraction of X‐rays by these crystals was still poor. After testing over 40 crystals, a 5.3‐Å SeMet single‐wavelength anomalous dispersion (SAD) dataset was collected at a wavelength of 0.97968 Å (the absorption edge of selenium) at beamline 14.2 of BESSY II.

All datasets were processed using the program *XDS* (Kabsch, 2010), and the crystals were nearly isomorphous in the native and SAD datasets. The space group was *P3_1_21*, with unit‐cell parameters *a* = *b* = 92.4 Å, *c* = 166.6 Å.

### Experimental phasing and model refinement

Using the Paip1M:SeMet‐Mac2 SAD data, the phases for this binary complex were finally determined by combining the SAD and MR methods. First, all four selenium positions of Mac2 (except that for the selenium atom from the extra N‐terminal GSHM residues remaining from the cloning vector) were successfully identified using *ShelxD* (Sheldrick, 2010), and the correct hand for the substructure was determined with *ShelxE* (Sheldrick, 2010). Then, the atomic coordinates of Mac2 were fixed according to the selenium positions, and Paip1M was located with the structure of the isolated protein (PDB entry: 3R6K, Lei *et al,* 2011) as search model (program *MOLREP*, Vagin & Teplyakov, 2010). On the basis of the resulting electron density map, an initial Mac2:Paip1M complex model was built using *Coot* (Emsley *et al*, 2010). This model was refined using BUSTER (Bricogne *et al*, 2017), complemented by model rebuilding rounds using *Coot*. Attempts to include higher‐resolution data did not lead to improvements of the electron density maps. The final refinement statistics are presented in Appendix Table S1.

### Small‐angle X‐ray scattering

Small‐angle X‐ray scattering (SAXS) measurements for Mac2, Paip1M, and Mac2:Paip1M were performed at the EMBL BioSAXS beamline P12 (Blanchet *et al*, 2015), DESY, Hamburg, Germany. A 2D photon‐counting Pilatus 2 M detector was used to record the scattered X‐rays. The sample‐to‐detector distance was 3.0 m. A series of different protein concentrations for each sample was prepared. The final concentrations of Mac2 were 5.6, 2.8, 1.3, 0.7, and 0.3 mg/ml. The concentrations of Paip1M were 16.2, 8.1, 4.1, 2.0, and 1.0 mg/ml, while the concentrations of the binary complex were 15.9, 7.9, 4.0, 2.0, and 1.0 mg/ml. All concentrations were determined by a NanoDrop ND‐1000 (Thermo Scientific). All samples were in buffer C with 2 mM DTT to avoid the formation of unspecific disulfide bonds at high protein concentrations. Then, all samples were measured with an X‐ray wavelength of 1.24 Å at 20°C.

The initial data were automatically processed by using the pipeline *SASFLOW* at DESY (Franke *et al*, 2012). The molecular masses (MW) of all scattering samples were obtained by the equation MW(BSA)/*I*_0_(BSA) = MW(X)/*I*_0_(X); X stands for the target protein, *I*_0_ is the intensity of zero‐angle scattering. A Guinier plot was used to evaluate the sample distribution. All Guinier plots were linear, indicating monodispersity of the sample solutions at every protein concentration. Subsequently, all SAXS measurement curves (five protein concentrations for each sample) were merged into one average curve for each sample by using the program *PRIMUSqt* (Konarev *et al*, 2003). The *ab‐initio* shape determination of each sample was carried out by using the program *DAMMIF* (Franke & Svergun, 2009).

### Isothermal titration calorimetry

The binding affinity between Paip1M and Mac2, Δ16‐Mac2, or Mac1–2 was determined at 25°C using a VP‐ITC titration calorimeter (MicroCal). The freshly purified Mac2, Δ16‐Mac2, or Mac1–2 was concentrated to 120 μM, and Paip1M was concentrated to 1,000 μM in buffer C.

About 2 ml Mac2, Δ16‐Mac2, or Mac1–2 was added into the sample cell. About 300 µl Paip1M was placed in the injection syringe for each ITC experiment. The dilution heat was measured by injecting Paip1M into the buffer alone, for control. The experiments were carried out by injecting Paip1M into solutions of Mac2, Δ16‐Mac2, or Mac1–2. The data obtained were then processed by using the MicroCal ORIGIN software.

### Micro‐scale thermophoresis‐binding assay

The His‐PABP‐pET28a plasmid was synthesized by General Biol (China). Expression and purification of His‐PABP protein and of Paip1 and SUD proteins without His‐tag was performed as described above. For the MST assay, all proteins were dissolved in 20 mM HEPES, 150 mM NaCl, 0.05% Tween‐20, pH 8.0 (named HEPES buffer in the following). Purified His‐PABP was labeled following the instructions of Monolith His‐Tag Labeling Kit RED‐tris‐NTA 2^nd^ Generation (NanoTemper Technologies) and then diluted in HEPES buffer at the final concentration of 50 nM. After 30 min incubation at room temperature, fluorophore‐labeled His‐PABP was centrifuged for 10 min at 4°C at 15,000 *g*. Paip1 was serially diluted with HEPES buffer starting from the highest concentration of 140 μM. Then, supernatant of fluorophore‐labeled PABP was added to Paip1 serial dilutions with equal volumes. These reaction samples were mixed completely by pipetting and loaded into standard capillaries for measurement at 22°C, 40% LED‐power, and medium MST‐power using a Monolith NT.115 instrument (NanoTemper Technologies). Data from two independent measurements of fluorescence signal corresponding to MST‐On time were analyzed using the MO. Affinity Analysis software (NanoTemper Technologies). His‐PABP:Paip1 samples with SUD (at the final concentration of 19 μM/tube) were measured at the same conditions. SUD (at the final concentration of 19 μM/tube) was added to His‐PABP as a control.

### Polyribosome gradient analysis

HEK‐293T cells were cultivated in Dulbecco's modified Eagle's medium (DMEM, Gibco) supplemented with 10% fetal bovine serum (FBS, Gibco), 1× penicillin/streptomycin (Gibco), and 1× GlutaMAX (Gibco). Plasmids pDEST‐c‐myc‐YFP^N^‐Paip1 and pDEST‐HA‐YFP^C^‐PABP, pDEST‐RFP‐SUD, pDEST‐RFP were transfected with Lipofectamine 3000 (ThermoFisher) following the manufacturer's protocol and HEK‐293T cells were grown for additional 40 h after transfection. Before harvesting, cycloheximide (100 µg/ml) was added to the media for 15 min at 37°C to prevent ribosome run‐off. Cells were washed with ice‐cold PBS supplemented with 100 µg/ml cycloheximide and detached from the cell culture dishes through scraping. Cells were resuspended in lysis buffer (20 mM Hepes‐KOH [pH 7.5], 100 mM KCl, 5 mM MgCl_2_, 1 mM DTT, 0.5% NP‐40, 100 µg/ml cycloheximide, 20 U/ml RNase inhibitor, and EDTA‐free protease inhibitor) and incubated for 5 min on ice. The lysate was passed six times through a 23‐gauge needle and cleared by centrifugation twice at 20,000 *g* for 10 min. Equivalent amounts of each sample (according to absorption at 260 nm) were loaded on linear 10–50% sucrose gradients (20 mM Hepes‐KOH [pH 7.5], 100 mM KCl, 5 mM MgCl_2_, 1 mM DTT, and EDTA‐free protease inhibitor), and samples were separated at 202,048 *g* for 2.5 h using a SW40 Ti rotor (Beckmann Coulter). The gradients were fractionated with a Biocomp piston gradient fractionator, and the absorption at 260 nm was measured with a Biocomp Triax flow cell. The fractions were precipitated with trichloroacetic acid and used for subsequent Western blot analysis.

### Plasmid constructs for *in vivo* assays

The fragments c‐myc‐YFP^N^ (aa 1–155) and HA‐YFP^C^ (aa 156–239) were amplified (using the primers listed in Appendix Table S2) from the template plasmids pSPYNE‐35S and pSPYCE‐35S (Walter *et al,* 2004), respectively. The obtained PCR fragments were subsequently cloned into the pTREX‐dest30‐PrA Gateway‐compatible vector (Pfefferle *et al,* 2011) via AgeI and XhoI cleavage sites or into the pTREX‐dest30‐PrA Gateway‐compatible vector via SpeI and ApaI sites to replace the protein A tag, yielding pDEST‐c‐myc‐YFP^N^ and pDEST‐HA‐YFP^C^ or pDEST‐ct‐c‐myc‐YFP^N^ and pDEST‐ct‐HA‐YFP^C^ vectors. SUD, Paip1, PABP, and the truncated sequences of SUD and Paip1 amplified with corresponding primers (Appendix Table S2) were first BP Gateway‐cloned into the pDONR207 vector and subsequently LR‐cloned into pDEST‐c‐myc‐YFP^N^/pDEST‐HA‐YFP^C^/pDEST‐ct‐c‐myc‐YFP^N^/pDEST‐ct‐HA‐YFP^C^ vectors, yielding constructs for the split‐YFP assays.

For the fluorescent‐3‐hybrid (F3H) assays, the Gateway rfB cassette was amplified with designed primers (Appendix Table S2) from the Gateway destination vector pTREX‐dest30‐PrA and then cloned into pNLS‐TagRFP and pNLS‐TagGFP vectors (Dambacher *et al*, 2012) via XhoI and HpaI sites, yielding pNLS‐TagRFP GW and pNLS‐TagGFP GW Gateway‐compatible vectors. Paip1, SUD, and their truncated sequences were finally cloned into pNLS‐TagRFP GW and pNLS‐TagGFP GW via the LR reaction.

For some of the luciferase activity assays and qPCR, SUD and its truncated constructs were cloned into the pDEST‐ct‐HA vector via the LR Gateway reaction. To examine the subcellular co‐localization of PABP, Paip1, and SUD, the RFP and GFP fragments without nuclear localization signal (NLS) were amplified with designed primers (Appendix Table S2) and subsequently cloned into pTREX‐dest30‐PrA via AgeI and XhoI sites, or into pTREX‐dest30‐ct‐PrA via SpeI and ApaI sites, to replace the protein A tag, yielding pDEST‐RFP and pDEST‐GFP or pDEST‐ct‐RFP and pDEST‐ct‐GFP destination vectors. PABP, Paip1, and SUD were cloned into pDEST‐RFP/GFP and pDEST‐ct‐RFP/GFP via the LR Gateway reaction. For the GFP‐trap‐based co‐immunoprecipitation assay, Paip1, Paip1M, and SUD constructs were cloned into pDEST‐ct‐HA and pDEST‐N‐c‐myc vectors via the LR Gateway reaction, respectively.

### Cells and transfection

HEK‐293 cells were cultured in Dulbecco's modified Eagle medium (DMEM) containing 10% FBS and 1% penicillin/streptomycin. Plasmids were transfected into cells by using Lipofectamine 3000 (Invitrogen) when the cells were 70% confluent.

For the split‐YFP assay, all related proteins were first checked in the Western blot (Appendix Fig S4B); then, cells were seeded directly on coverslips and cultured in 24‐well plates. Pictures were subsequently taken with a Leica DM4000 B fluorescence microscope 24 h after transfection.

For the Luciferase Activity Assay and qPCR, HEK‐293 cells were seeded on 96‐well plates. 5 ng pRL Renilla Luciferase control reporter together with 200 ng SUD, Mac2, or Δ16‐Mac2 expressing plasmid DNA was co‐transfected to each well. Cells were harvested 24 h post‐transfection for further measurement.

For the fluorescence‐3‐hybrid assay, BHK cells containing lac operator repeats (Tsukamoto *et al*, 2000) were seeded on coverslips and cultured in DMEM with 10% FCS. TagGFP‐ and TagRFP‐fused constructs were transfected into the cells together with the GBP‐LacI construct (Rothbauer *et al*, 2006) using polyethylenimine (Sigma). About 16 h later, cells were fixed with 3.7% formaldehyde for 10 min and then washed three times with PBST (PBS with 0.02% Tween) and stained with DAPI (200 ng/ml) to show the nucleus. Samples were mounted in Vectashield medium (Vectors Laboratories). Samples were analyzed using a laser scanning confocal microscope TCS SP5 (Leica) with a 63×/1.4 NA oil immersion objective as described (Dambacher *et al*, 2012). A 488‐nm argon laser was used to illuminate TagGFP. DAPI and TagRFP were excited by a 405‐nm diode laser and a 561‐nm diode‐pumped solid‐state laser, respectively. Digital images were recorded by a PMT detector with a frame size of 512 × 512 pixels.

### Luciferase activity assay

Cells were washed once with PBS and put on ice. Subsequently, 40 µl/well of Renilla Lysis Buffer (Promega) were added. After 20 min of shaking on ice, the lysate was transferred to a 96‐well white polystyrene microplate (Greiner Bio‐One). The luciferase activity was measured with a FLUOstar OPTIMA‐Fluorescence plate reader (BMG Labtech). Experiments were performed in at least triplicates.

### Ribopuromycylation assay

The ribopuromycylation assay was carried out as described elsewhere (Schmidt *et al,* 2009). HEK‐293 cells growing in a 12‐well plate were transfected with the indicated plasmids and replicon via Lipofectamine 3000 (Invitrogen). Twenty‐four hours post‐transfection, the cells were pulsed with 3 µM puromycin for 1 h at 37°C before harvesting for Western blot analysis.

### Probe‐based qPCR

RNA was extracted with the ISOLATE RNA Mini Kit (Bioline) and quantified by real‐time PCR using SensiFAST™ Probe Hi‐ROX One‐Step Kit (Bioline) allowing reverse transcription and PCR amplification in a single step. The real‐time PCR was performed in a Roche LightCycler 96. The Renilla luciferase, β‐actin, and pBAC‐SARS‐CoV replicon qPCR primers are listed in Appendix Table S2. Experiments were performed in at least triplicates.

### SYBR Green qPCR

RNA was extracted with the ISOLATE RNA Mini Kit (Bioline), and 1^st^ cDNA was isolated with SuperScript™ IV Reverse Transcriptase Kit (Invitrogen). The SYBR green qPCR was carried out by using AceQ qPCR SYBR® Green Master Mix Kit (Vazyme Biotech) in a Roche LightCycler 96.

### Co‐immunoprecipitation and Western blotting

pDEST‐GFP‐PABP or pDEST‐GFP, pDEST‐Paip1‐HA or pDEST‐Paip1M‐HA, and pDEST‐c‐myc‐SUD were co‐transfected into HEK‐293 cells growing in 10‐cm cell culture dishes. Cells were harvested 48 h post‐transfection. GFP‐Trap®_A beads for immunoprecipitation of GFP‐Fusion Proteins (ChromoTek) were used to pull down the complex of GFP‐PABP via its standard protocol under native conditions. The eluted samples and total cell lysates were then analyzed by Western blotting. The performed Western blotting protocol was described elsewhere (Carbajo‐Lozoya *et al*, 2014).

### Antibodies used for Western blots

Anti‐HA: Invitrogen (Cat. No. 26183, 1:10,000 dilution), anti‐GFP: Invitrogen, (Cat. No. A‐6455, 1:1,000 dilution), anti‐RFP: Invitrogen, (Cat. No. MA5‐15257, 1:1,000 dilution), anti‐PABP: Sigma‐Aldrich, (Cat. No. P6246, 1:2,000 dilution), anti‐puromycin: (Millipore, [Cat. No. MABE341, 1:100,000 dilution], anti‐vinculin: Sigma‐Aldrich, [Cat. No. V9264, 1:1,000 dilution], anti‐β‐actin: Santa Cruz, [Cat. No. sc‐47778, 1:500 dilution], anti‐myc: Roche [9E10], 1:300 dilution).

## Author contributions

JL, YM‐L, AvB, and RH designed experiments. JL performed SAXS, ITC, gel filtration assays, and crystallized the complex and determined its structure. YH and JL conducted MST experiments. YM‐L conducted split‐YPF, CoIP, qPCR, Western blot, and luciferase activity assays. MT, RBu, and RBe contributed polyribosome gradient experiments. VT and JJ provided the SARS‐CoV‐2 cDNA. WD and HL performed the F3H assay. JL, YM‐L, AvB, and RH wrote the manuscript.

## Conflict of interest

The authors declare that they have no conflict of interest.

## Supporting information



Appendix

Expanded View Figures PDF

Review Process File

## Data Availability

Atomic coordinates and structure factors for the Mac2:Paip1M complex have been deposited in the Protein Data Bank with accession code 6YXJ (https://www.rcsb.org/structure/6YXJ).
