# Supplementary material for: The SARS‐unique domain (SUD) of SARS‐CoV and SARS‐CoV‐2 interacts with human Paip1 to enhance viral RNA translation
Source: EMBO J. 2021 Apr 20;40(11):e102277. doi: 10.15252/embj.2019102277 (PMC8167360; doi:10.15252/embj.2019102277)
Supplement: Supplementary file 1 — Appendix [file EMBJ-40-e102277-s002.pdf]

Appendix

Appendix tables and figures

APPENDIX FIGURES

Appendix Figure S1. SUD and Paip1 interaction confirmed by fluorescence-3-hybrid (F3H) assay.

Appendix Figure S2. Testing various SUD-related constructs for interaction with Paip1M *in vitro*.

Appendix Figure S3. The N-terminal 16 residues of Mac2 are involved in binding to Paip1M and Mac1 cannot affect Mac2:Paip1M binding.

Appendix Figure S4. Luciferase mRNA level checking and western blot assay.

APPENDIX TABLES

Appendix Table S1. Diffraction data collection and refinement statistics.
Appendix Table S2. Results of the SAXS experiments
Appendix Table S3. Primers.

Appendix Fig. S1

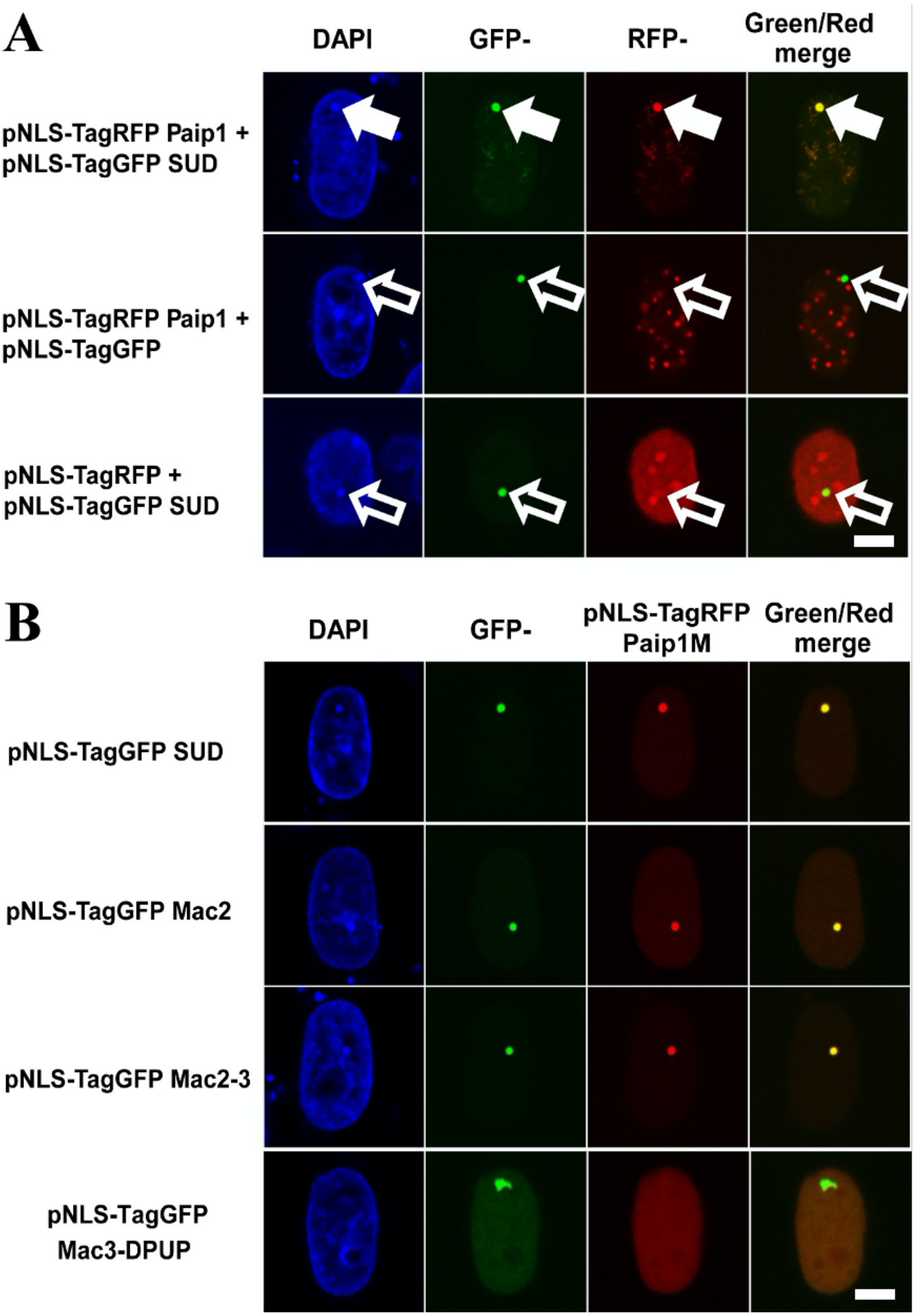

Appendix Fig. S1. SUD and Paip1 interaction confirmed by fluorescence-3-hybrid (F3H) assay. (A) The SUD interaction with Paip1 in BHK cells was demonstrated by

the F3H assay described previously (Herce *et al*, 2013). NLS-GFP-tagged SUD captured at the *lacO* site by the co-expressed GBP-LacI fusion is visualized as a green spot *via* fluorescence microscopy. Paip1 tagged with NLS-RFP co-localizes with SUD at the *lacO* spot (pointed out by white arrows), indicating an interaction between these two proteins. Using the absence of SUD (middle) or Paip1 (bottom) as negative controls, the RFP fusion targets were not recruited to the *lacO* spot (empty arrows). The nucleus was stained with DAPI. NLS, nuclear localization sequence; *lacO*, lac operator; GBP, GFP-binding protein. LacI, *lac* repressor. (B) Mac2 is essential for Paip1M binding in the F3H assay. Scale bars represent 5 µm.

Appendix Fig. S2

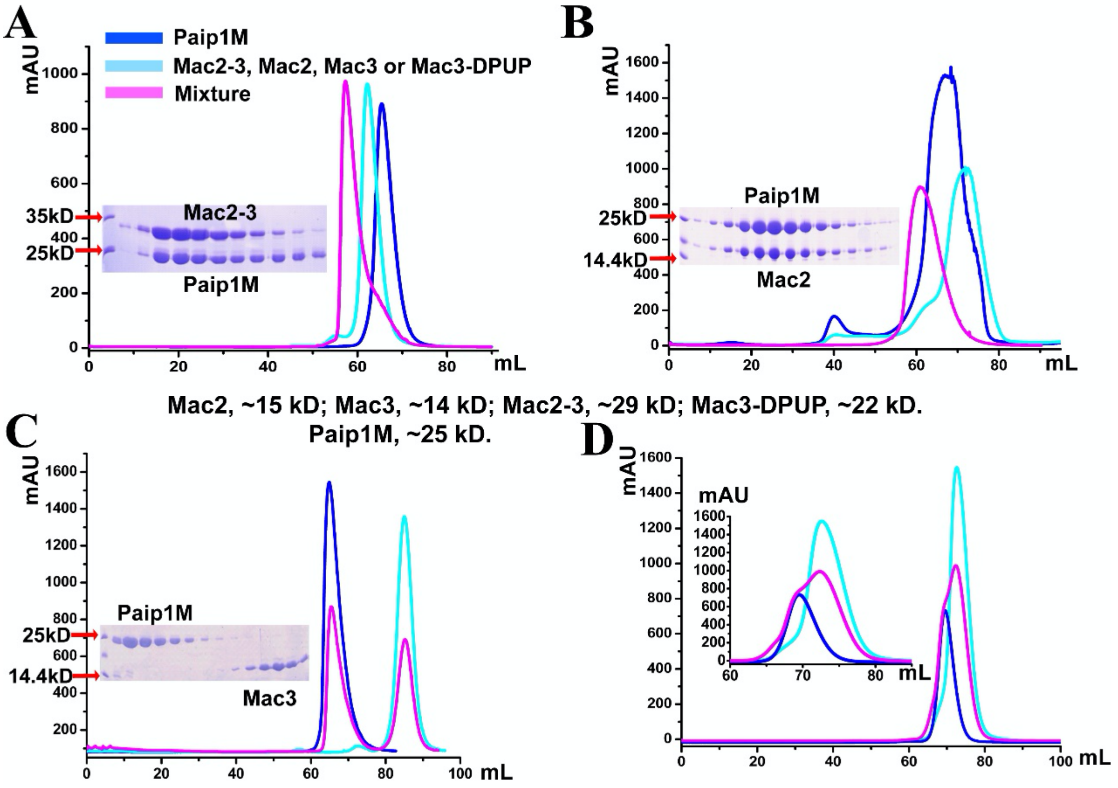

Appendix Fig. S2. Testing various SUD-related constructs for interaction with Paip1M *in vitro*. The mixtures of Mac2–3 (A) as well as Mac2 (B) with Paip1M show an obvious peak shift compared to the single peak of each protein, whereas Mac3 (C) or Mac3–DPUP (D) do not bind to Paip1M in the gel-filtration assay.

Appendix Fig. S3

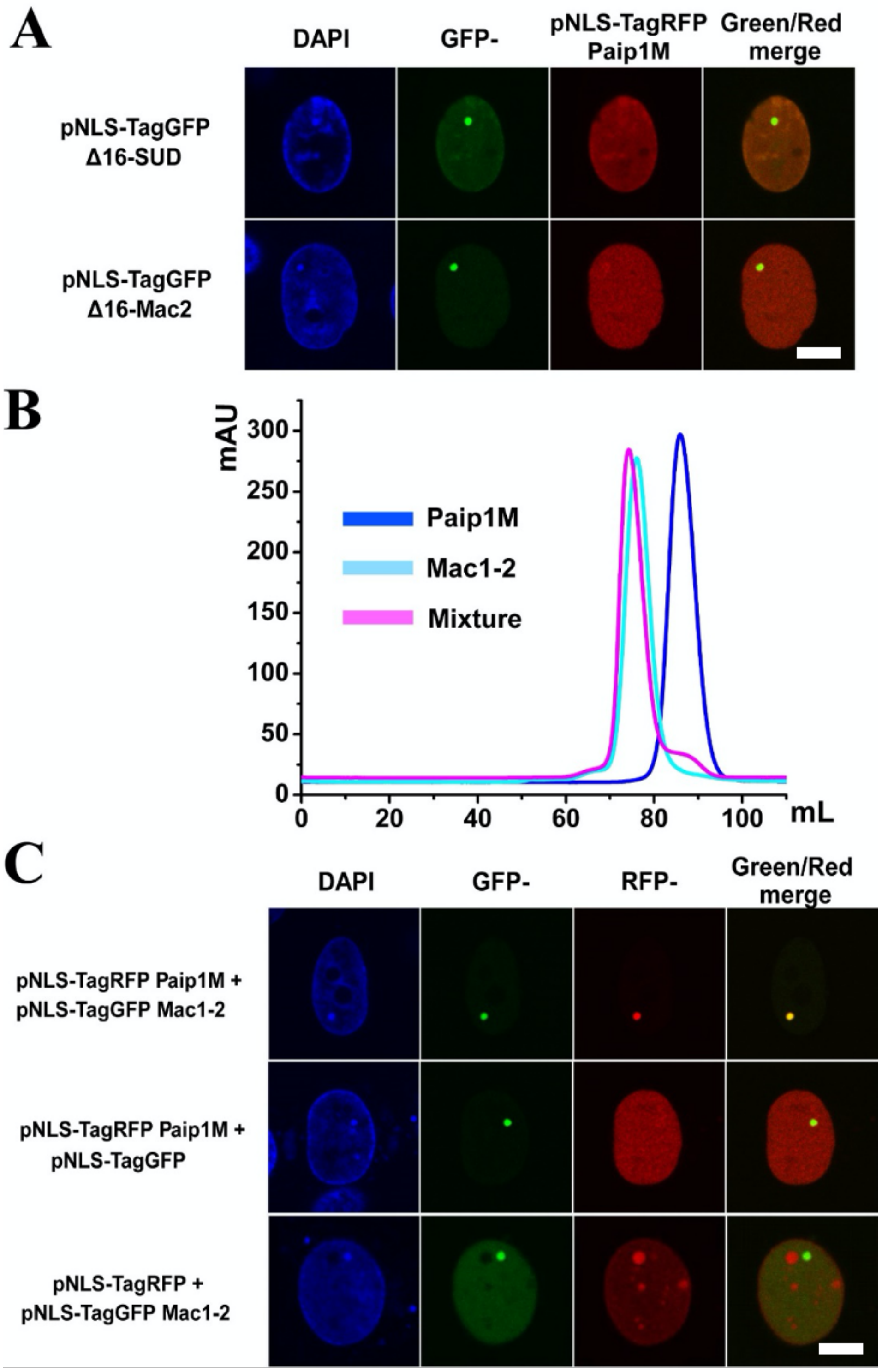

Appendix Fig. S3. The N-terminal 16 residues of Mac2 are involved in binding to Paip1M and Mac1 cannot affect Mac2:Paip1M binding. (A) Δ16-SUD or Δ16-Mac2 cannot interact with Paip1M in BHK cells in the F3H assay. (B) Mac1–2 and Paip1M form a complex in the gel-filtration assay. (C) The interaction between Mac1–2 and Paip1M was confirmed in BHK cells by the F3H assay. Scale bars represent 5 µm.

Appendix Fig. S4

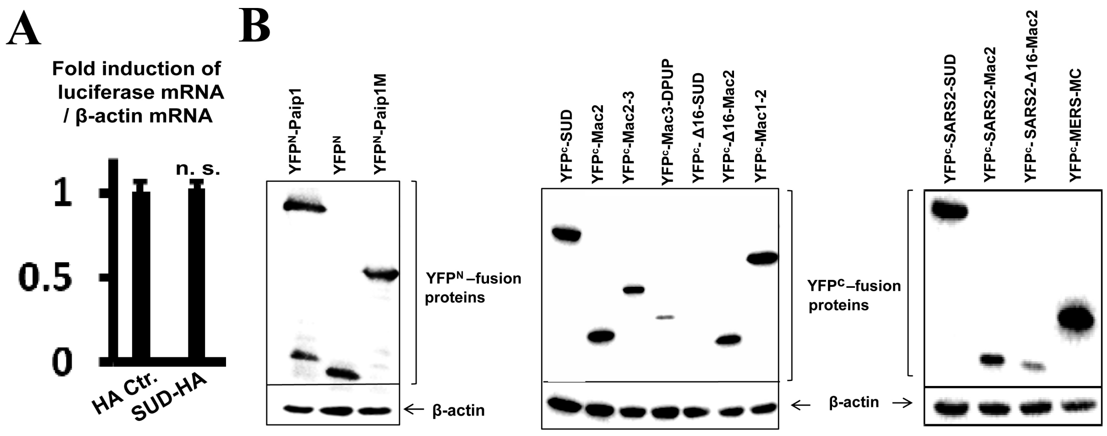

Appendix Fig. S4. Luciferase mRNA level checking and western blot assay. (A) The luciferase mRNA level does not change between co-transfected HA and SUD-HA plasmids. A luciferase-pcDNA3 reporter plasmid with HA and SUD-HA plasmids were transfected into HEK-293 cells, respectively. Cells were harvested 24 hours post-transfection to extract total RNA for q-PCR. The ratio of luciferase mRNA to β-actin mRNA was calculated for analyzing the induction. (B) The expression amount of split-YFP fusion proteins. HEK-293 cells growing in 6-well plates were transfected with the indicated plasmids. 24 hours post-transfection, total protein was harvested for western blot.

Appendix Table S1. Diffraction data collection and refinement statistics

	Crystal types	
	Native	Se (peak point)
Data collection statistics		
Space group	*P*3_1 2 1	*P*3_1 2 1
Unit-cell dimensions (Å, °)	$a = b = 92.4$, $c = 166.6$	$a = b = 92.7$, $c = 167.2$
	α = β = 90, γ = 120	α = β = 90, γ = 120
Wavelength (Å)	0.91840	0.97968
*V*m (Å^3/Da)	4.76	4.76
Solvent content (%)	74.2	74.2
Resolution range (Å)	46.18-3.50 (3.69-3.50)	45.76-5.30 (5.59-5.30)
Number of unique reflections	10784 (1549)	3289 (464)
Completeness (%)	99.3 (99.7)	99.7 (99.8)
R_{merge}‡	0.095 (1.250)	0.073 (0.920)
R_{pim}§	0.043 (0.547)	0.022 (0.263)
Mean I/σ(I)	10.8 (1.8)	14.6 (2.9)
Multiplicity	5.9 (6.0)	12.3 (12.9)
$CC_{1/2}$¶	0.999 (0.753)	0.999 (0.983)
Wilson B-factor (Å^2)	132	
Phasing statistics		
SHELXD CCall (%)*		60.43
SHELXD CCweak (%)*		30.85
SHELXD CFOM**		91.27
Heavy atom sites		4 Se
Refinement statistics		
R_{factor} (%)	31.3	
R_{free} (%)#	33.6	
Protein atoms	2726	
r.m.s. deviation in bond lengths (Å)	0.008	
r.m.s. deviation in bond angles (°)	0.97	
Average *B*-factor for all atoms (Å^2)	255	
Residues in favoured regions (%)	96.0	
Residues in allowed regions (%)	3.0	
Residues in outlier regions (%)	1.0	

‡ $R_{merge} = \sum_{hkl}\sum_i |I_i(hkl) - \langle I(hkl)\rangle| / \sum_{hkl}\sum_i I_i(hkl)$.

§ $R_{pim} = \sum_{hkl} \{1/[N(hkl) - 1]\}^{1/2} \times \sum_j |I_i(hkl) - \langle I(hkl)\rangle| / \sum_{hkl}\sum_j I_i(hkl)$ (Weiss & Hilgenfeld, 1997).

¶ $CC_{1/2}$ is the correlation coefficient determined by two random half data sets (Karplus & Diederichs, 2012).

*CC: Correlation coefficient, the value measures the reliability of the solution. CC_{all} is the CC between the normalized structure factor differences in measured data and those calculated from a given substructure solution; CC_{weak} is the same, but only for weak reflections which have not been used for direct methods (a somewhat independent criterion) (Thorn, 2017). For SAD, a CC of more than 30% is a safe sign of a correct solution.
**CFOM: Combined figure of merit, which is CCall + CCweak. (Please visit the website shelx.uni-goettingen.de for more information)
$R_{factor} = \sum_{hkl} | F_o(hkl) - F_c(hkl) | / \sum_{hkl} F_o(hkl)$. R_{free} was calculated for a test set of reflections (7.95%) omitted from the refinement.

Appendix Table S2. Results of the SAXS experiments

	BSA	Mac2	Paip1M	Mac2:Paip1M
I_0	0.05 ± 4.3 x 10^{-5}	0.01 ± 4.6 x 10^{-5}	0.02 ± 2.2 x1 0^{-5}	0.03 ± 1.9 x 10^{-5}
Rg (Å)	29 ± 3	17 ± 1	24 ± 1	26 ± 4
Rg* (Å)		14	19	23
MW (kDa)		13.2 ± 0.1	26.4 ± 0.03	39.6 ± 0.03
MW† (kDa)	66	15.6	26.6	42.2

* Rg values calculated from crystal structures; † Theoretical molecular mass.

Appendix Table S3.

Primers used for *in-vitro* assays

SUD forward	5′-GGAATTCCATATG AAAATTAAGGCCTGCATTGATGAGGTTAC-3′
SUD backward	5′-CCGCTCGAGTTAGGATAAGAGACTCTTTAGTTTGTCAAGTG-3′
Mac2–3 forward	5′-GGAATTCCATATGAAAATTAAGGCCTGCATTGATGAGGTTAC-3′
Mac2–3 backward	5′-CCGCTCGAGTTATGATGACGAAGTGAGGTATCCATTATATG-3′
Mac2 forward	5′-GGAATTCCATATGAAAATTAAGGCCTGCATTGATGAGG-3′
Mac2 backward	5′-CCGCTCGAGTTATAGAATCTCTTCCTTAGCATTAGG-3′
Mac3 forward	5′-GGAATTCCATATGGGAACTGTATCCTGGAATTTGAGAGAAATG-3′
Mac3 backward	5′-CCGCTCGAGTTATGATGACGAAGTGAGGTATCCATTATATG-3′
Mac3–DPUP forward	5′-GGAATTCCATATGGGAACTGTATCCTGGAATTTGAGAGAAATG-3′
Mac3–DPUP backward	5′-CCGCTCGAGTTAGGATAAGAGACTCTTTAGTTTGTCAAGTG-3′
Δ16 Mac2 forward	5′-GGAATTCCATATGAAGTTTCTTACCAATAAGTTACTC-3′
Δ16 Mac2 backward	5′-CCGCTCGAGTTATAGAATCTCTTCCTTAGCATTAGG-3′
Mac1–2 forward	5′-CGCGGATCCGAAGAACCAGTTAATCAGTTTACTG-3′
Mac1–2 backward	5′-CCGCTCGAGTTATAGAATCTCTTCCTTAGCATTAGG-3′

Primers used for *in-vivo* assays

c-myc YFP^{N}(a.a.1-155) forward	5'-CGCTCACCGGTACTAGTATGGAGCAAA AGTTGATTTC-3'
c-myc YFP^{N}(a.a.1-155) backward	5'-CTCTGGGCCCTTAACTCGAGAAGGCCATGATATAG CGTTG-3'
HA YFP^{C}(a.a.156-239) forward	5'-GAGAGACCGGTACTAGTATGTACCCATACGATGTT C-3'
HA YFP^{C}(a.a.156-239) backward	5'-ATAAGGGCCCTTAACTCGAGAACTTGTACAGCTCGTCCATG-3'
Paip1 forward	5'-GGGGACAAGTTTGTACAAAAAAGCAGGCTCCGCCATGGCTAAGCCCCA GGTGGTTGTAGC-3'
Paip1 backward with stopcodon	5'-GGGGACCACTTTGTACAAGAAAGCTGGGTCTTACTGTTTTCGCTTACGCTCTGATTCC-3'
Paip1 backward without stopcodon	5'-GGGGACCACTTTGTACAAGAAAGCTGGGTCTCCCTGTTTTCGCTTACG CTCTGATTCC-3'
Paip1M forward	5'-GGGGACAAGTTTGTACAAAAAAGCAGGCTCCGCCATGACTCTATCAGAATATGTTCAGGATTTTTGAATC-3'
Paip1M backward	5'-GGGGACCACTTTGTACAAGAAAGCTGGGTCTCMACTTGACCGGAGTTCTACAAGCTTCAAGAG-3'
SUD/Mac2/Mac2–3 forward	5'-GGGGACAAGTTTGTACAAAAAAGCAGGCTCCGCCATGAAAATTAAGGC CTGCATTGATGAGG-3'
SUD/Mac3–DPUP/Δ16-SUD backward	5'-GGGGACCACTTTGTACAAGAAAGCTGGGTCTCMGGATAAGAGACTCTTTAGTTTGTCAAGTGAAAGAAC-3'
Mac2/Δ16-Mac2/Mac1–2 backward	5'-GGGGACCACTTTGTACAAGAAAGCTGGGTCTCMTAGAATCTCTTCCTTAGCATTAGGTGCTTC-3'
Mac2–3 backward	5'-GGGGACCACTTTGTACAAGAAAGCTGGGTCTCMTGATGACGAAGTGAGGTATCCATTATATGTAG-3'
Mac3–DPUP forward	5'-GGGGACAAGTTTGTACAAAAAAGCAGGCTCCGCCATGAATTTGAGAGAAATGCTTGCTCATGCTGAAG-3'
Δ16-Mac2/Δ16-SUD forward	5'-GGGGACAAGTTTGTACAAAAAAGCAGGCTCCGCCATGAAGTTTCTTAC CAATAAGTTACTCTTG-3'

Mac1–2 forward	5'-GGGGACAAGTTTGTACAAAAAAGCAGGCTCCGCCATGGAAGAACCAGTTAATCAGTTTACTGG-3'
PABP forward	5'-TTGGACAAGTTTGTACAAAAAAGCAGGCTCCGCCATGAACCCCAGTGC CCCCAGCTACCCCATGG-3'
PABP backward	5'-GGGGACCACTTTGTACAAGAAAGCTGGGTCTCM^{II}AACAGTTGGAACACC GGTGGCACTGTTAAC-3' (M^{II}: A or C)
rfB cassette forward	5'-CTCAGATCTCGAGGGATATCAACAAGTTTGTAC-3'
rfB cassette backward	5'-CTGGCGTTAACGATATCAACCACTTTGTACAAG-3'
RFP forward	5'-CGCTCACCGGTACTAGTATGGTGTCTAAGGGCGAAGAGC-3'
RFP backward	5'-CTCTGGGCCCTTAACTCGAGAAATTAAGTTTGTGCCCCAG-3'
GFP forward	5'-TATAGACCGGTACTAGTATGAGCGGGGGCGAGGAGCTGTTC-3'
GFP backward	5'-ATAAGGGCCCTTAACTCGAGAACAGCTCGTCCATGCCGTGG-3'
Renilla luc. forward	5'-TATACAGCGAGAAGCACGCCGAGAAC-3'
Renilla luc. backward	5'-ATATCCACTCGTCCCAGCTCTCGATC-3'
Renilla luc. probe	5'-Fam-ACTACAAGTACCTGACCGCCTGG-BHQ-1-3'
β-actin forward	5'-ATATAGGCCGTCTTCCCCTCCATCG-3'
β-actin backward	5'-ATGGAGTCCATCACGATGCCAGTG-3'
β-actin probe	5'-Fam-GCATCCTCACCCTGAAGTACCCCATC-BHQ-1-3'
SARS Replicon qPCR nsp14 forward	5'-CCCGCGAAGAAGCTATTCG-3'
SARS Replicon qPCR nsp14 reverse	5'AGTTGCATGACAGCCCTCTACA-3'
SARS Replicon qPCR nsp14 probe	5'-Fam-ACGTTCGTGCGTGGATTGGCTTTG-BHQ-1-3'
RFP SYBR green qPCR forward	5'-CGCTCACCGGTACTAGTATGGTGTCTAAGGGCGAAGAGC-3'
RFP SYBR green qPCR reverse	5'-CTCGACCACCTTGATTCTC-3'
SARS-2 SUD/Mac2 forward	5'-GGGGACAAGTTTGTACAAAAAAGCAGGCTCCGCCATGAAAATCAAAGCTTGTGTTGAAGAAGTTAC-3'

SARS-2 SUD/Δ16-SUD reverse	5’-GGGGACCACTTTGTACAAGAAAGCTGGGTCTCMAGAAAGAAGTGTCTTAAGATTGTCAAAGG-3’
SARS-2 Δ16Mac2/Δ16-SUD forward	5’-GGGGACAAGTTTGTACAAAAAAGCAGGCTCCGCCATGAAGTTCCTCACAGAAAACTTGTTACTTTATATTG-3’
SARS-2 Mac2/ Δ16Mac2 reverse	5’-GGGGACCACTTTGTACAAGAAAGCTGGGTCTCMAATTTCTTGCTTCTCATTAGAGATAATAGATGGTAG-3’
MERS-MC forward	5'-GGCGACAAGTTTGTACAAAAAAGCAGGCTTCGCCATGATTCCACAGAGTTTGACTTTTTCATATGATGG-3‘
MERS-X-MC forward	5'-GGCGACAAGTTTGTACAAAAAAGCAGGCTTCGCCATGGACCCTTTGTCCAATTTTGAACATAAGG-3‘
MERS-MC/X-MC reverse	5'-GAGGACCACTTTGTACAAGAAAGCTGGGTCTCMCTGCTGTGTCGTGCGTGAATCCAAATACGCACG-3‘

Appendix References

Blanchet CE, Spilotros A, Schwemmer F, Graewert MA, Kikhney A, Jeffries CM, Franke D, Mark D, Zengerle R, Cipriani F *et al* (2015) Versatile sample environments and automation for biological solution X-ray scattering experiments at the P12 beamline (PETRA III, DESY). *J Appl Crystallogr* 48: 431 – 443

Bricogne G, Blanc E, Brandl M, Flensburg C, Keller P, Paciorek W, Roversi P, Sharff A, Smart OS, Vonrhein C *et al* (2017) BUSTER version 2.10.3. Cambridge, United Kingdom: Global Phasing Ltd.

Carbajo-Lozoya J, Ma-Lauer Y, Malešević M, Theuerkorn M, Kahlert V, Prell E, von Brunn B, Muth D, Baumert TF, Drosten C, Fischer G,

von Brunn A (2014) Human coronavirus NL63 replication is cyclophilin A-dependent and inhibited by non-immunosuppressive cyclosporine A-derivatives including Alisporivir. *Virus Res* 184: 44 – 53

Dambacher S, Deng W, Hahn M, Sadic D, Fröhlich J, Nuber A, Hoischen C, Diekmann S, Leonhardt H, Schotta G (2012) CENP-C facilitates the recruitment of M18BP1 to centromeric chromatin. *Nucleus* 3: 101 – 110

Emsley P, Lohkamp B, Scott WG, Cowtan K (2010) Features and development of *Coot*. *Acta Crystallogr D Biol Crystallogr* 66: 486 – 501

Franke D, Kikhney AG, Svergun DI (2012) Automated acquisition and analysis of small angle x-ray scattering data. *Nuc Inst Meth A* 689: 52 – 59

Franke D, Svergun DI (2009) *DAMMIF*, a program for rapid ab-initio shape determination in small-angle scattering. *J Appl Crystallogr* 42: 342 – 346

Herce HD, Deng W, Helma J, Leonhardt H, Cardoso MC (2013) Visualization and targeted disruption of protein interactions in living cells. *Nat Commun* 4: 2660

Kabsch W (2010) *XDS*. *Acta Crystallogr D Biol Crystallogr* 66: 125 – 132

Karplus PA, Diederichs K (2012) Linking crystallographic model and data

quality. *Science* 336: 1030 – 1033

Konarev PV, Volkov VV, Sokolova AV, Koch MHJ, Svergun DI (2003) *PRIMUS*: a Windows PC-based system for small-angle scattering data analysis. *J Appl Crystallogr* 36: 1277 – 1282

Lei J, Kusov Y, Hilgenfeld R (2018) Nsp3 of coronaviruses: Structures and functions of a large multi-domain protein. *Antiviral Res* 149: 58 – 74

Lei J, Mesters JR, von Brunn A, Hilgenfeld R (2011) Crystal structure of the middle domain of human poly(A)-binding protein-interacting protein 1. *Biochem Biophys Res Commun* 408: 680 – 685

Pfefferle S, Schöpf J, Kögl M, Friedel CC, Müller MA, Carbajo-Lozoya J, Stellberger T, von Dall'Armi E, Herzog P, Kallies S *et al* (2011) The SARS-coronavirus-host interactome: identification of cyclophilins as target for pan-coronavirus inhibitors. *PLoS Pathog* 7: e1002331

Rothbauer U, Zolghadr K, Tillib S, Nowak D, Schermelleh L, Gahl A, Backmann N, Conrath K, Muyldermans S, Cardoso MC *et al* (2006) Targeting and tracing antigens in live cells with fluorescent nanobodies. *Nat Methods* 3: 887 – 889

Schmidt EK, Clavarino G, Ceppi M, Pierre P (2009) SUnSET, a nonradioactive method to monitor protein synthesis. *Nat Methods* 6: 275 – 277

Sheldrick GM (2010) Experimental phasing with *SHELXC/D/E*: combining chain tracing with density modification. *Acta Crystallogr*

D Biol Crystallogr 66: 479 – 485

Tan J, Vonrhein C, Smart OS, Bricogne G, Bollati M, Kusov Y, Hansen G, Mesters JR, Schmidt CL, Hilgenfeld R (2009) The SARS-unique domain (SUD) of SARS coronavirus contains two macrodomains that bind G-quadruplexes. *PLoS Pathog* 5: e1000428

Thao TTN, Labroussaa F, Ebert N, V'kovski P, Stalder H, Portmann J, Kelly J, Steiner S, Holwerda M, Kratzel A *et al* (2020) Rapid reconstruction of SARS-CoV-2 using a synthetic genomics platform. *Nature* 582: 561-565.

Thorn A. (2017) Experimental Phasing: Substructure Solution and Density Modification as Implemented in SHELX. In: Wlodawer A., Dauter Z., Jaskolski M. (eds) Protein Crystallography. Methods in Molecular Biology, vol 1607. Humana Press, New York, NY. https://doi.org/10.1007/978-1-4939-7000-1_15.

Tsukamoto T, Hashiguchi N, Janicki SM, Tumbar T, Belmont AS, Spector DL (2000) Visualization of gene activity in living cells. *Nat Cell Biol* 2: 871 – 878

Vagin A, Teplyakov A (2010) Molecular replacement with *MOLREP*. *Acta Crystallogr D Biol Crystallogr* 66: 22 – 25

Walter M, Chaban C, Schütze K, Batistic O, Weckermann K, Näke C, Blazevic D, Grefen C, Schumacher K, Oecking C *et al* (2004) Visualization of protein interactions in living plant cells using

bimolecular fluorescence complementation. *Plant J* 40: 428 – 438

Weiss MS, Hilgenfeld R (1997) On the use of the merging *R* factor as a quality indicator for X-ray data. *J Appl Crystallogr* 30: 203 – 205
