# Supplementary material for: The SARS‐unique domain (SUD) of SARS‐CoV and SARS‐CoV‐2 interacts with human Paip1 to enhance viral RNA translation
Source: EMBO J. 2021 Apr 20;40(11):e102277. doi: 10.15252/embj.2019102277 (PMC8167360; doi:10.15252/embj.2019102277)
Supplement: Supplementary file 2 — Expanded View Figures PDF [file EMBJ-40-e102277-s003.pdf]

Expanded View Figures

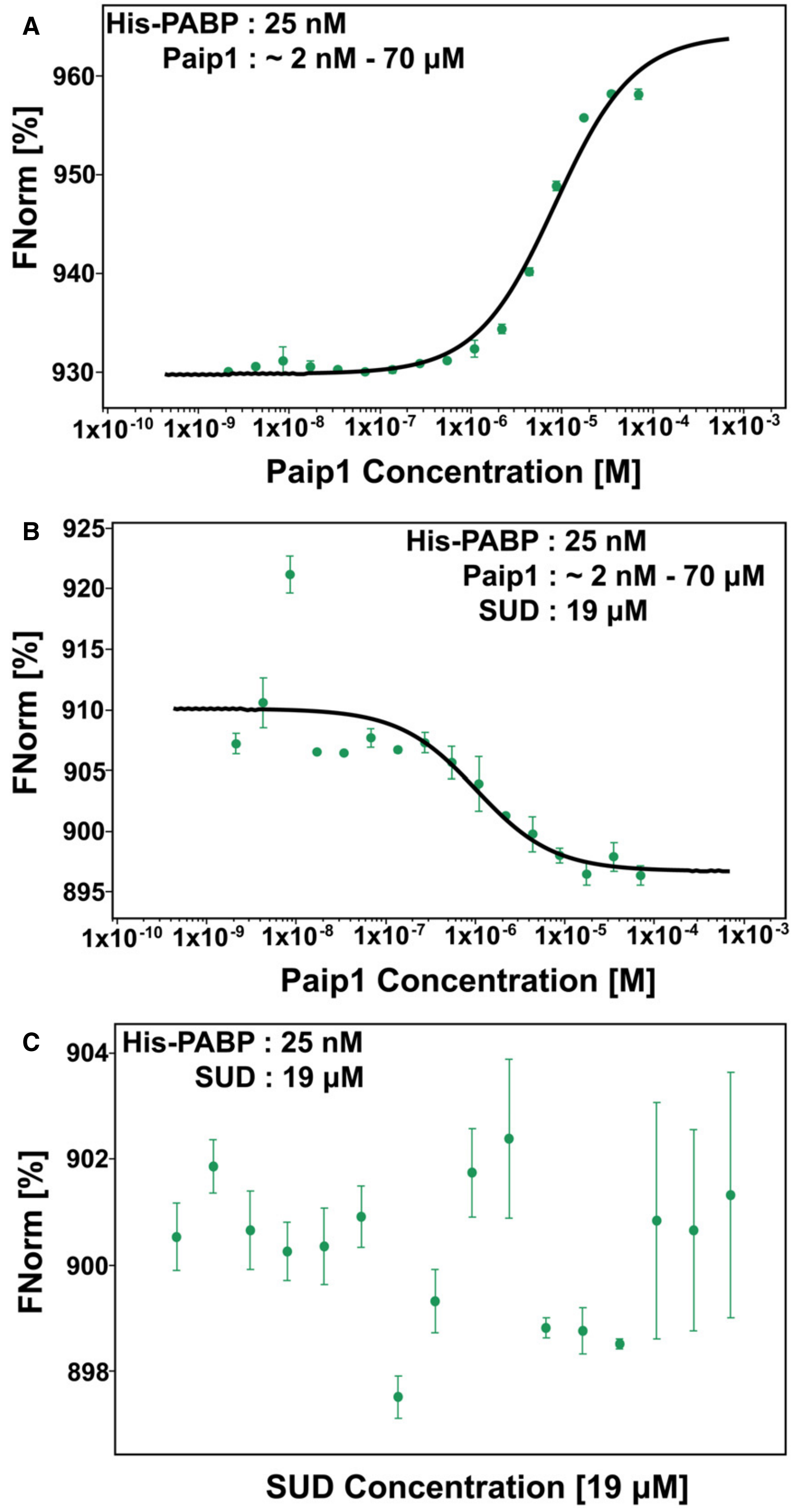

Figure EV1. SUD enhances the interaction between PABP and Paip1.

A Curve fit of micro-scale thermophoresis (MST) traces for the determination of the Paip1 and PABP-binding affinity (K_d = 8.4 ± 1.5 µM).

B Curve fit of MST traces for determination of the Paip1 and PABP-binding affinity upon adding SUD (K_d = 1.9 ± 0.6 µM).

C Control: SUD and PABP display no binding affinity.

Data information: Data are shown as the mean ± SD from two independent replicates.

 © 2021 The Authors

Figure EV2. Polyribosome gradient analysis of lysates from HEK293T cells transiently transfected with either pDEST-RFP-SUD/pDEST-c-myc-YFP^N-Paip1/ pDEST-HA-YFP^C-PABP or pDEST-RFP/pDEST-c-myc-YFP^N-Paip1/pDEST-HA-YFP^C-PABP constructs.

The absorption at 260 nm (A_{260}) and the collected fractions are shown and 40S, 60S, 80S, and polyribosome fractions are labeled. Western blot analysis of the gradient fractions using anti-RFP, anti-c-myc (c-myc-Paip1 fusion), anti-PABP, and rps6 (S6, 40S ribosomal subunit protein) antibodies is shown below.

© 2021 The Authors

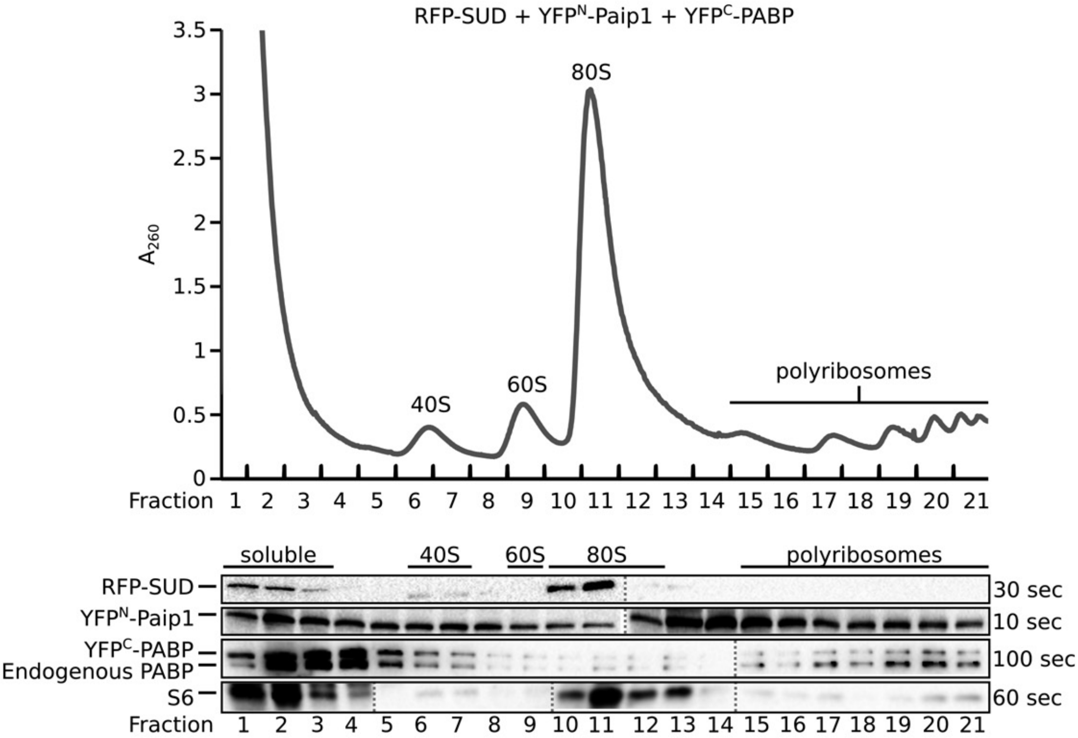

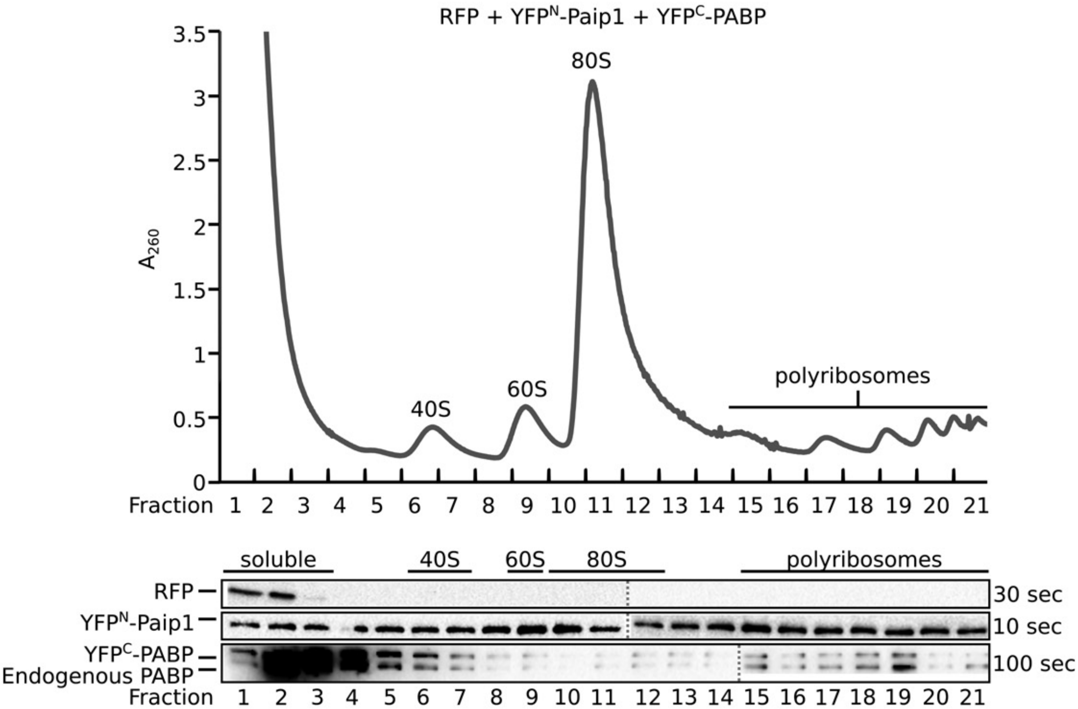

Figure EV2.

© 2021 The Authors

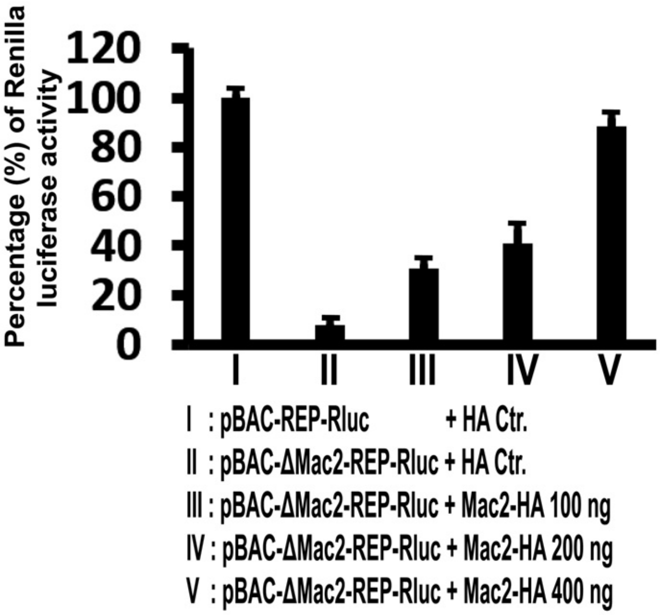

Figure EV3. Increasing dose of Mac2 leads to increasing rescue of Renilla luciferase activity from pBAC-ΔMac2-REP-RLuc.

The indicated replicons and plasmids were transfected into HEK293 cells using Lipofectamine 3000. Twenty-four hours post-transfection, cells were lysed and Renilla luciferase activity of the lysate was measured using Promega Renilla luciferase assay kit E2820. Percentage of Renilla luciferase activity was calculated as ratio of luciferase activity of pBAC-ΔMac2-REP-RLuc to pBAC-REP-RLuc. Error bars represent SD ($n = 6$).

© 2021 The Authors
